# Metal Ion‐Induced Fast Gelation of 2D MnO_2_ Nanosheets With Cationic Vacancies for Durable Aqueous Zinc‐Ion Batteries

**DOI:** 10.1002/advs.76281

**Published:** 2026-06-25

**Authors:** Yalei Wang, Xinyu Huang, Shulong Chang, Bowen Li, Caichao Ye, Feng Yang, Yahui Xue

**Affiliations:** ^1^ Department of Mechanics and Aerospace Engineering & Center for Complex Flows and Soft Matter Research Southern University of Science and Technology Shenzhen China; ^2^ Guangdong Provincial Key Laboratory of Sustainable Biomimetic Materials and Green Energy Southern University of Science and Technology Shenzhen China; ^3^ School of Advanced Manufacturing and Robotics Peking University Beijing China; ^4^ Department of Chemistry Southern University of Science and Technology Shenzhen China; ^5^ Academy For Advanced Interdisciplinary Studies and Department of Materials Science and Engineering Guang Dong Provincial Key Laboratory of Computational Science and Material Design Southern University of Science and Technology Shenzhen China

**Keywords:** 3D assembly, 3D MnO_2_ aerogel, aqueous zinc‐ion storage, gelation, vacancy engineering

## Abstract

Delta manganese dioxide (δ‐MnO_2_) has been extensively investigated as an attractive cathode material for aqueous zinc‐ion batteries (AZIBs) owing to its large interlayer spacing that facilitates ion storage and transport. However, the random restacking of 2D MnO_2_ nanosheets significantly limits their accessible surface area and increases diffusion resistance, thereby severely degrading electrochemical performance. Herein, we report a scalable synthesis of 3D structured aerogels from defect‐rich MnO_2_ nanosheets via a divalent metal ion‐initiated self‐assembly process for durable aqueous zinc‐ion batteries. The addition of metal ions enables MnO_2_ nanosheets in solution to assemble into a 3D network by neutralizing electrostatic repulsion and serving as intersheet linkers. The obtained 3D Mn vacancy‐rich MnO_2_ aerogel (A‐MnO_2_) cathode demonstrate greatly enhanced electrochemical performance, especially superior rate performance (146.3 mAh g^−1^ at 5 A g^−1^), and long‐term cycling stability (90.6% capacity retention after 5000 cycles at 5 A g^−1^). Moreover, the experimental analysis and theoretical calculations reveal that A‐MnO_2_ delivers improved conductivity, fast reaction kinetics, and superior structural stability, owing to abundant Mn vacancies and a 3D porous structure. This work may provide new insight for guiding the structural design of 2D electrode materials in AZIBs.

## Graphical abstract

We report Mn‐deficient aerogels by inducing the first gelation of MnO_2_ nanosheets in solution using divalent metal ions as crosslinking agents, thereby inhibiting nanosheet restacking and enhancing surface utilization. Benefiting from abundant Mn vacancies and 3D porous structures, the A‐MnO_2_ cathode exhibits improved conductivity, fast reaction kinetics, and superior structural stability.

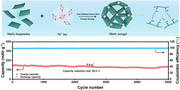

## 1 Introduction

Large‐scale battery systems are urgently needed to store the renewable energy and help mitigate the global energy crisis [1]. Although lithium‐ion batteries (LIBs) are one of the most mature energy storage systems, their large‐scale application is increasingly limited by resource scarcities, safety concerns, and rising costs [2]. In contrast, aqueous zinc ion batteries (AZIBs) have been considered as a prospective candidate for next‐generation large‐scale energy storage in terms of the abundant Zn resource, inherent safety, and low cost [3]. Among the key components in ZIBs, the cathode materials usually provide active sites for ion storage and largely determine the electrochemical performances of batteries [4]. To date, a series of cathode materials such as manganese‐based oxides [5], vanadium‐based oxides [6], metal chalcogenides [7], Prussian blue analogues [8], and organic compounds [9] have been reported. Among these materials, MnO_2_ has emerged as a particularly attractive cathode owing to its low cost, environmental friendliness, high theoretical capacity, and suitable operating voltage [10]. In particular, δ‐MnO_2_, a layered structure with an interlayer spacing of ∼7.0 Å, can avoid the phase transition and provide abundant active sites during the electrochemical reaction, thereby maintaining structural integrity and facilitating ion storage and transport [11, 12, 13]. However, similar to other two‐dimensional (2D) materials, the random restacking of MnO_2_ nanosheets leads to a pronounced loss of accessible surface area and increased ion diffusion resistance, severely degrading their specific capacity and rate performance. Besides, MnO_2_ suffers from intrinsically poor electrical conductivity, which also hampers its electrochemical performance [14]. Therefore, the rational structural design is urgently needed to address these issues.

Some reports suggest that integrating 2D materials into three‐dimensional (3D) macroscopic architectures is regarded as an important and effective strategy to address the restacking issue [15, 16]. Such 3D architectures featuring controlled morphologies can deliver large accessible surface areas and interconnected networks, simultaneously suppressing the undesirable restacking of 2D materials and thus achieving high‐performance electrodes [17]. The gelation of 2D nanosheets is the most commonly employed approach to construct 3D assembled architectures, which is induced by surface chemistry variations of 2D nanosheets dispersed in solution. However, previous reports on the gelation of 2D materials have primarily focused on GO and MXene nanosheets, owing to their good solution processability. Typically, a 3D MXene‐based aerogel using graphene oxide (GO) as the linker has been fabricated via solution‐phase assembly, exhibiting enhanced performance in photocatalytic, environmental cleaning, and energy storage fields [18, 19]. The gelation of GO and MXene nanosheets can also be realized using other linkers, such as surfactants, polymers, and metal ions [20, 21, 22, 23, 24]. Notably, metal ions as linkers are particularly effective in modulating the electronic bandgap and stabilizing the crystal structure [25]. For other 2D materials, such as transition metal oxides, transition metal dichalcogenides, and h‐BN, the gelation is difficult to achieve due to their limited surface functional groups and poor dispersion in aqueous solutions. To date, the gelation of MnO_2_ nanosheets using metal ion crosslinking strategies similar to those employed for GO and MXene remains challenging and has not yet been reported to the best of our knowledge.

Furthermore, defect engineering enables precise modulation of local atomic structures and coordination environments, leading to improved electron and ion transport [26]. Reported defect engineering strategies for MnO_2_ in ZIBs primarily involve cation vacancies, anion vacancies, cationic doping, and anionic doping. As a representative type of cation vacancy, Mn vacancies can weaken the repulsive forces and modulate charge distribution in the lattice, thereby facilitating ion transfer kinetics and enhancing electronic conductivity. For instance, Chao et al. proposed an innovative electrolytic Zn‐MnO_2_ system and used density functional theory (DFT) to explore the role of Mn vacancies in regulating MnO_2_ behavior during the electrochemical reaction. Surface electron density difference analysis indicates increased electron density at Mn vacancies, which is conducive to the reaction kinetics by lowering the energy barrier on the potential energy surface [27]. In addition, the presence of Mn vacancies can create extra sites for ion intercalation, leading to improved electrochemical activity. For instance, V_Mn_‐Mn_3_O_4_ is designed by integrating Mn vacancies with a Mn‐ion confinement effect, thereby effectively improving the electrochemical performance of Mn_3_O_4_. The introduction of Mn vacancies provides more electrochemically active sites, resulting in an enhanced specific capacity [28]. In light of the above analyses, 3D MnO_2_ aerogels incorporating cation vacancies offer a viable and effective approach for synergistically tailoring the cathode structure in AZIBs.

Herein, 3D Mn vacancy‐rich MnO_2_ aerogels (A‐MnO_2_) have been synthesized via a self‐assembly approach using Ni^2+^ ions as a linker for durable aqueous zinc ion battery applications. Mn vacancies were generated during the exfoliation process through the extraction of interlayer K^+^ ions, together with the disproportionation of Mn^3+^ ions. Moreover, the addition of Ni^2+^ ions effectively screens the electrostatic repulsion between MnO_2_ nanosheets, enabling their crosslinking into a stable 3D hydrogel, similar to the gelation mechanisms observed in GO and MXene hydrogels. Furthermore, unlike conventional porous MnO_2_ fabrication approaches that typically rely on sacrificial templates or conductive composite scaffolds, the proposed ion‐induced gelation strategy directly utilizes the surface chemistry of MnO_2_ nanosheets and divalent‐ion crosslinking interactions to achieve 3D assembly. Specifically, the unique 3D interconnected framework with increased Mn vacancies, enabled by Ni^2+^ crosslinking, provides (i) enlarged electrolyte‐accessible surface area, (ii) rapid electron/ion transport pathways, and (iii) enhanced structural stability during repeated ion insertion/extraction. As expected, the resultant aerogels suppress MnO_2_ nanosheet restacking and consequently exhibit outstanding electrochemical performance. A‐MnO_2_ cathode delivers a high specific capacity of 308.3 mAh g^−1^ at 0.1 A g^−1^ and a stable reversible capacity of 146.3 mAh g^−1^ with 90.6% capacity retention after 5000 cycles at 5 A g^−1^. Electrochemical measurements combined with DFT calculations further demonstrate that the unique structural design endows A‐MnO_2_ with improved electronic conductivity, reflected by a reduced bandgap of 0.61 eV, as well as rapid ion transport kinetics with diffusion coefficients ranging from 10^−^^7^ to 10^−12^ cm^2^ s^−1^.

## 2 Results and Discussion

### 2.1 Synthesis and Morphological Characterization

**FIGURE 1:**
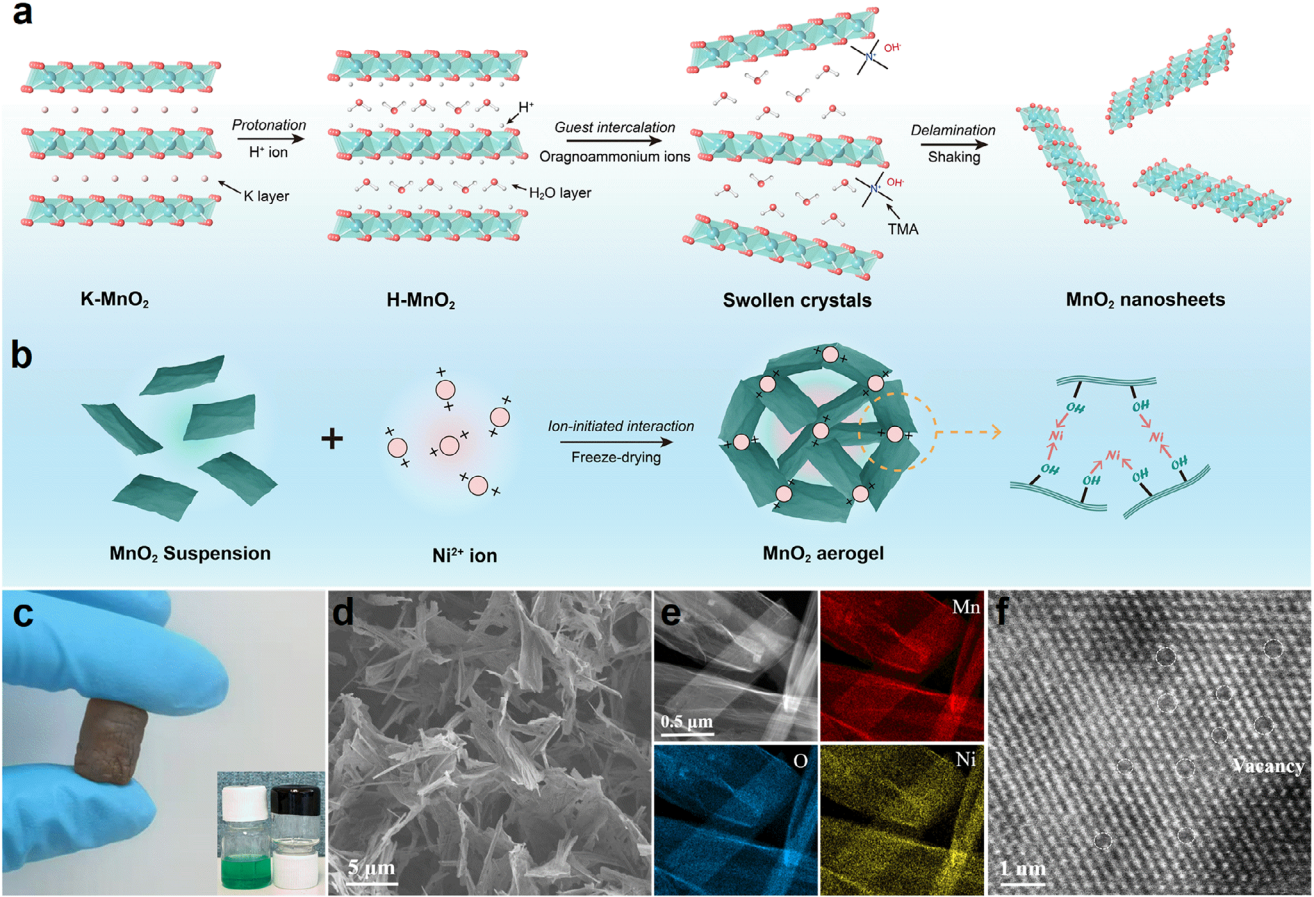
The synthesis and morphological characterization of the MnO_2_ aerogel. (a) The exfoliation process of pristine MnO_2_ nanosheets. (b) Synthesis diagram of the MnO_2_ aerogel. (c) Digital image (inset: optical photograph of MnO_2_ after mixing with divalent Ni^2+^ ions), (d) SEM image, (e) Elemental mapping images, and (f) HAADF‐STEM image of the MnO_2_ aerogel.

The MnO_2_ nanosheets were obtained by a typical top‐down liquid exfoliation approach (Figure 1a). Note that the thickness of the MnO_2_ nanosheets is measured as 0.92 nm (Figure S1), in good accordance with the values of unilamellar MnO_2_ nanosheets covered by modified functional groups [29, 30]. The exfoliated MnO_2_ nanosheets are terminated with ─OH functional groups (Figure S2). Thus, MnO_2_ nanosheets can be dispersed in water to form stable colloidal dispersions because of the electrostatic repulsion [20, 31]. The MnO_2_ aerogel (A‐MnO_2_) was designed and synthesized through the electrostatic self‐assembly and freeze‐drying technique (Figure 1b). Typically, the MnO_2_ dispersion (10 mg mL^−1^) was added to NiCl_2_ solutions (1 m) and the hydrogel formed within several seconds, manifesting the generation of 3D interconnected networks. Then, A‐MnO_2_ was obtained by the freeze‐drying (Figure 1c). In a typical procedure, the hydrophilic functional groups enable the uniform dispersion of MnO_2_ nanosheets in water through electrostatic repulsion between adjacent nanosheets. Ni^2+^ ions are then introduced into the MnO_2_ dispersion to weaken the electrostatic repulsion and bridge neighboring nanosheets, thereby inducing the formation of a stable 3D MnO_2_ hydrogel. The metal ions act as effective crosslinking sites through their strong interactions with the surface ─OH groups of MnO_2_, promoting the construction of an interconnected 3D network. As shown in Figure S3, the decreased intensity of the ─OH peak in the Fourier transform infrared (FTIR) spectra after gelation suggests an interaction between the ─OH groups and Ni^2+^ ions, which is believed to contribute significantly to the enhanced structural integrity and stability of the gel framework. In general, metal ions play an important role in the gelation of MnO_2_ nanosheets. The inset shows an optical photograph of MnO_2_ after mixing with divalent Ni^2+^ ions. Through experiments, other metal ions (K^+^ and Al^3+^) have also been attempted to induce the gelation process. However, univalent K^+^ ions with poor hydration energy only cause coagulation without the formation of MnO_2_ hydrogels (Figure S4). In contrast, the MnO_2_ hydrogel with the assistance of trivalent Al^3+^ ions shows the severe aggregation, which is possibly due to the fact that the electrostatic repulsion between MnO_2_ nanosheets was rapidly disrupted by trivalent ions with more positive charges (Figure S5). Therefore, divalent Ni^2+^ ions are more suitable for triggering the gelation of MnO_2_ nanosheets. Scanning electron microscope (SEM), energy dispersive x‐ray (EDX) element mapping, and high‐angle annular dark‐field scanning transmission electron microscopy (HAADF‐STEM) were utilized to investigate the morphology and microstructure of A‐MnO_2_. From the SEM image (Figure 1d and Figure S6), A‐MnO_2_ shows 3D interlinked networks composed of ultrathin MnO_2_ nanosheets, which can facilitate the transmission of ions and provide sufficient active sites for electrochemical reactions [32, 33]. In contrast, the direct freeze‐drying of pristine MnO_2_ dispersions causes severe self‐stacking (Figure S7). Additionally, the elemental mappings (Figure 1e) exhibit the uniform distribution of Mn, O, and N elements in A‐MnO_2_. Moreover, the HAADF‐STEM image of A‐MnO_2_ shows hazy atomic distributions in dotted circles, proving the existence of Mn vacancies (Figure 1f).

### 2.2 Structural Characterizations

**FIGURE 2:**
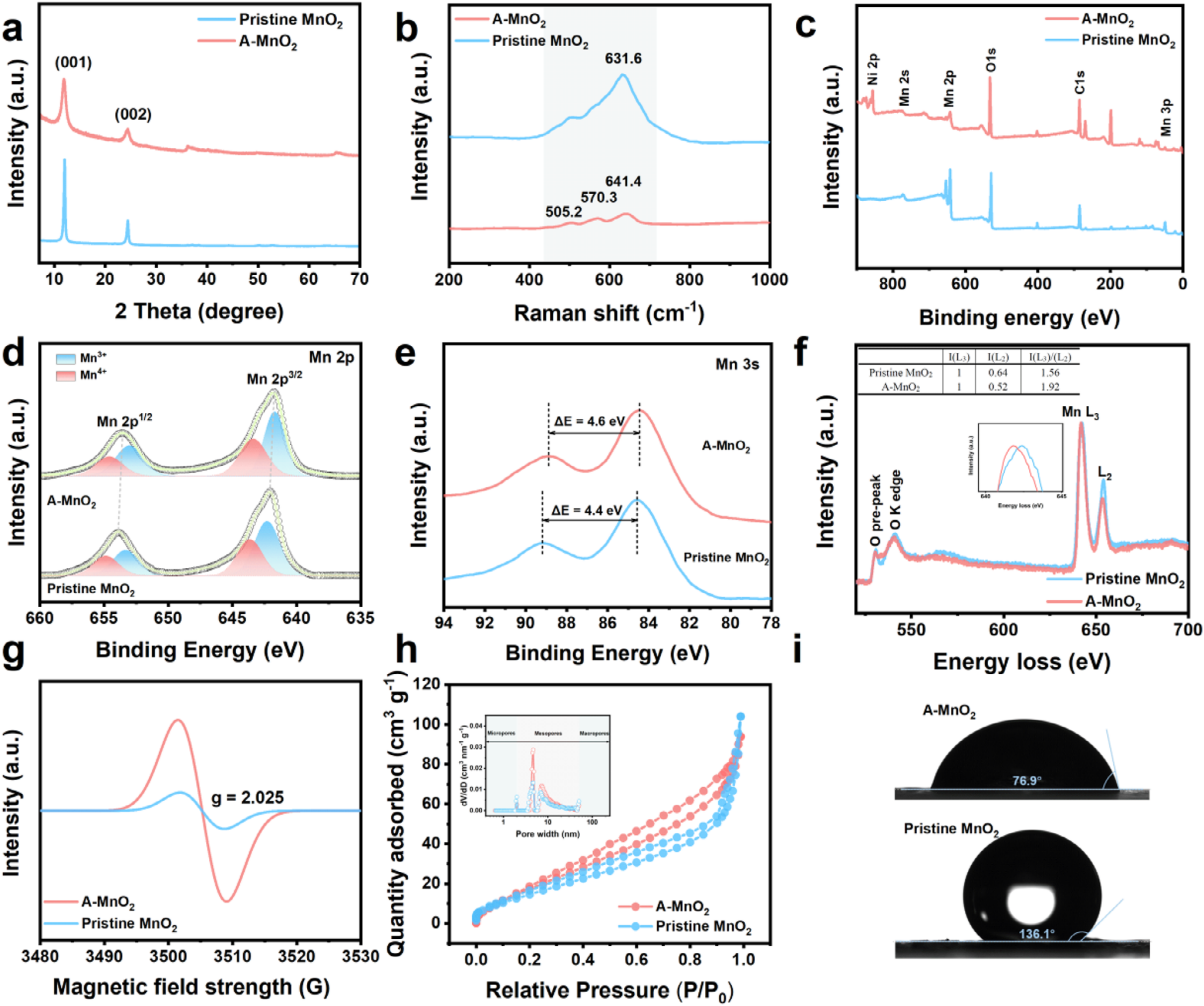
Structural characterizations of pristine MnO_2_ and A‐MnO_2_. (a) XRD patterns. (b) Raman spectra. (c) XPS survey spectra. (d) Mn 2p XPS spectra. (e) Mn 3s XPS spectra. (f) EELS patterns (inset table: Mn white‐line intensity ratios of pristine MnO_2_ and A‐MnO_2_). (g) EPR curves. (h) Nitrogen adsorption‐desorption isotherms (inset: pore size distribution). (i) Electrolyte contact angle tests.

X‐ray diffraction (XRD) was applied to study the crystal structures of samples. As shown in Figure 2a, pristine MnO_2_ and A‐MnO_2_ show almost identical XRD patterns, which agree well with the δ‐MnO_2_ (JCPDS No. 80–1098), proving that the introduction of Ni^2+^ ions does not change the crystal phase of pristine MnO_2_. Notably, the broadening and weakening of (001) and (002) peaks can be observed after introducing Ni^2+^ ions into pristine MnO_2_, which may be attributed to the existence of defects in A‐MnO_2_ crystal structure [34, 35]. Raman analysis was further utilized to evaluate the impact of Ni^2+^ ions on the crystal structure of pristine MnO_2_ in Figure 2b. There are three characteristic peaks located at about 505.2 (*v*_3_), 570.3 (*v*_2_), and 641.4 cm^−1^(*v*_1_) for A‐MnO_2_, corresponding to the stretching vibration of Mn─O─Mn and Mn─O bonds in the basal plane of MnO_6_ units, and the symmetric stretching vibration of Mn−O bond in [MnO_6_] groups, respectively [36]. Compared with pristine MnO_2_, the *v*_1_ vibration of A‐MnO_2_ shifts toward a higher wavenumber, which is related to the decreased symmetry of the [MnO_6_] octahedra [37]. Moreover, the significant reduction in *v*_2_ and *v*_3_ peak intensities suggests the creation of numerous Mn vacancies and the weakening of Mn−O vibration along the chains of the framework [38].

The chemical composition and Mn vacancy of pristine MnO_2_ and A‐MnO_2_ were confirmed by x‐ray photoelectron spectroscopy (XPS) spectra. Through the full spectrum analysis (Figure 2c), A‐MnO_2_ is composed of Mn, O, C, and Ni elements (Figure S8). Moreover, compared to pristine MnO_2_ (642.1 eV for Mn 2p^3/2^ and 653.9 eV for Mn 2p^1/2^), the Mn 2p peaks of A‐MnO_2_ (641.8 eV for Mn 2p^3/2^ and 653.6 eV for Mn 2p^1/2^) slightly shift to the direction of lower binding energy (Figure 2d), presumably due to surface Mn vacancies as well as the charge compensation from the replacement of some Mn^4+^ within the nanosheets by Mn^3+^, which leads to a decrease in the average oxidation state (AOS) [39]. Meanwhile, the AOS of Mn can be calculated using the formula AOS = 8.95–1.13ΔE [40], where ΔE represents the energy difference between the main Mn 3s peak and its satellite peak. Based on calculations, the AOS of Mn decreases from 3.97 (pristine MnO_2_) to 3.75 (A‐MnO_2_), indicating a reduction in the oxidation state of Mn (Figure 2e). The presence of Mn vacancy was further verified by electron energy‐loss spectroscopy (EELS) and electron paramagnetic resonance (EPR) analysis. EELS signals show O K‐edges and Mn L‐edges (Figure 2f). The Mn white‐line intensity ratio is the ratio of the areas of the Mn L3 and L2 peaks in the deconvoluted spectra, which can be used as a measure of Mn valence (ratio increases as the oxidation state decreases) [41]. As shown in the insert table in Figure 2f, the Mn white‐line intensity ratio of A‐MnO_2_ (1.92) is greater than that of pristine MnO_2_ (1.56), indicating a lower Mn oxidation state in A‐MnO_2_. Simultaneously, the Mn L‐edges of A‐MnO_2_ show a negative shift to the lower energy loss position, further proving the decrease of Mn valence state in A‐MnO_2_. The reduction of the Mn valence state is due to the substitution of some Mn^4+^ by Mn^3+^ compensating for the charge change and simultaneously forming substantial Mn vacancies. The EELS analysis results were consistent with the XPS analysis results, both indicating the formation of a large number of Mn vacancies. In addition, from the EPR spectra in Figure 2g, the signal of A‐MnO_2_ at g = 2.025 is significantly stronger than that of pristine MnO_2_, demonstrating the increase in the concentration of Mn vacancies within A‐MnO_2_ [42]. Therefore, there are more Mn vacancies in A‐MnO_2_ compared to pristine MnO_2_, which plays a crucial role in promoting the transport of ions/electrons.

Brunauer‐Emmett‐Teller (BET) measurements were employed to characterize specific surface areas and pore size distributions of pristine MnO_2_ and A‐MnO_2_. As shown in Figure 2h, pristine MnO_2_ and A‐MnO_2_ exhibit comparable mesoporous size distributions, while their respective BET specific surface areas (SSA) are determined to be 58.2 m^2^ g^−1^ and 78.8 m^2^ g^−1^, respectively. The larger SSA and richer mesoporous structure exhibited by A‐MnO_2_ can offer more active sites and facilitate better interface contact between the electrolyte and electrode material, thereby achieving higher capacity and faster ion diffusion [43]. More importantly, the more mesopores of A‐MnO_2_ can buffer the large volume change caused by ion insertion/extraction during the electrochemical reaction process, thus enhancing the cycle life of the battery [44]. The contact angle measurement was used to investigate the effects of electrolyte on electrode wettability. Notably, A‐MnO_2_ electrode displays a contact angle of 76.9° (Figure 2i), indicating better wettability compared to pristine MnO_2_ in ZnSO_4_ electrolyte, which is beneficial for improving electrochemical performance.

### 2.3 Electrochemical Performance

**FIGURE 3:**
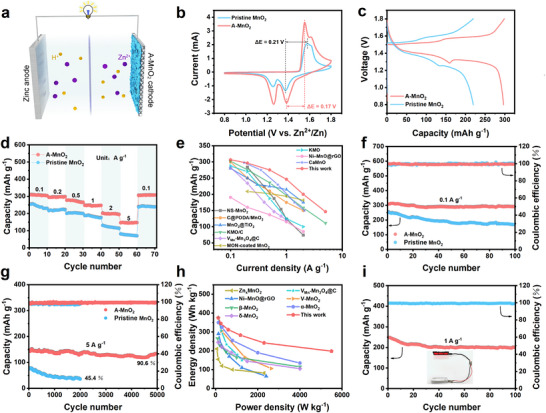
The electrochemical performance of pristine MnO_2_ and A‐MnO_2_ cathodes. (a) Configuration of the ZIBs with A‐MnO_2_ cathode and Zn anode. (b) CV curves at a scan rate of 0.5 mV s^−1^. (c) Galvanostatic charge/discharge profiles at 0.2 A g^−1^. (d) Rate performance at different current densities. (e) Rate performance of A‐MnO_2_ compared with reported cathode materials. (f) Cycling performance at 0.1 A g^−1^. (g) Long‐term cycling performance at 5 A g^−1^. (h) Comparison of energy density and power density. (i) Cycling performance of A‐MnO_2_‐based pouch cell at 1 A g^−1^ (inset: A‐MnO_2_‐based pouch cell powering LEDs).

To evaluate the advantage of A‐MnO_2_ structural design, the prepared samples are fabricated as cathodes to assemble coin cells (Figure 3a). Cyclic voltammetry (CV) curves recorded at a scan rate of 0.5 mV s^−1^ exhibit two pairs of distinct redox peaks, corresponding to the valence state change of Mn accompanied by reversible ion insertion/extraction reactions (Figure 3b). Notably, compared with pristine MnO_2_, the A‐MnO_2_ cathode demonstrates larger CV areas and lower voltage hysteresis, implying higher capacity and faster reaction kinetics [45]. Meanwhile, the galvanostatic charge/discharge (GCD) curves (Figure 3c) at 0.2 A g^−1^ display that A‐MnO_2_ cathodes possess wider voltage platforms and lower polarizations relative to the pristine MnO_2_, which is in good agreement with the CV results. These collectively suggest that metal ion crosslinking improves the activity of electrochemical reactions and promotes electrode kinetics [46]. Then, the rate performance of pristine MnO_2_ and A‐MnO_2_ cathodes at various current densities is compared, and the results are displayed in Figure 3d. A‐MnO_2_ cathode exhibits discharge capacities of 308.3, 296.6, 275.3, 246.5, 200.1, and 146.3 mAh g^−1^ at 0.1, 0.2, 0.5, 1, 2, and 5 A g^−1^, respectively (Figure S9). When the current density returns back to 0.1 A g^−1^, the discharge capacity can still restore to 308.1 mAh g^−1^ (Figure 3d), demonstrating A‐MnO_2_ cathodes possess high reversibility and structural stability. Notably, the rate performance of A‐MnO_2_ cathode is significantly better than that of pristine MnO_2_ cathode and most reported Mn‐based cathodes (Figure 3e and Figure S10) [6, 28, 47, 48, 49, 50, 51, 52, 53]. The cycling performance of A‐MnO_2_ cathode was also evaluated. As displayed in Figure 3f, A‐MnO_2_ shows a high discharge capacity of 290.3 mAh g^−1^ after 100 cycles at 0.1 A g^−1^, and there is no significant change in the morphology (Figure S11). In contrast, the discharge capacity of pristine MnO_2_ rapidly decreases to 169.8 mAh g^−1^ with only 67.4% capacity retention after 100 cycles. Even at 5 A g^−1^, A‐MnO_2_ delivers a relatively high discharge capacity of 146.3 mAh g^−1^ and retains 90.6% initial capacity after 5000 cycles with the coulombic efficiency (CE) approaches 99.8% (Figure 3g). In contrast, pristine MnO_2_ shows a low capacity of 35.9 mAh g^−1^ with only 45.4% capacity retention after 2000 cycles. Correspondingly, SEM images indicate that A‐MnO_2_ preserves its morphology after 5000 cycles (Figure S12), while pristine MnO_2_ undergoes significant structural collapse after 2000 cycles (Figure S13).

We further investigated the influence of Ni^2+^ content on the electrochemical performance by tuning the NiCl_2_/MnO_2_ ratio. As shown in Figure S14, the long‐term cycling performance gradually deteriorated as the NiCl_2_/MnO_2_ mass ratio increased from 1:2 to 4:5. SEM and the corresponding elemental mapping images are shown in Figures S15–S17. At the high mass ratio of 4:5, severe aggregation of MnO_2_ nanosheets was observed (Figure S17), which can be attributed to the excessive ionic strength at high NiCl_2_ concentrations, resulting in rapid electrostatic screening and destabilization of the MnO_2_ nanosheet dispersion. We additionally evaluated the electrochemical performance of the A‐MnO_2_ electrode in an Mn^2+^‐free electrolyte system. As shown in Figure S18, although the cycling stability and capacity retention are lower than those in the Mn^2+^‐containing electrolyte, the A‐MnO_2_ electrode still exhibits better electrochemical stability than the pristine MnO_2_ electrode. This indicates that the enhanced performance does not solely originate from the Mn^2+^ electrolyte additive, but is also closely associated with the unique 3D porous architecture and Ni^2+^‐mediated crosslinked framework with abundant Mn vacancies. To further clarify the role of the 3D porous structure in electrochemical performance, a non‐aerogel MnO_2_ sample (non‐A‐MnO_2_) was prepared by directly drying the Ni^2+^‐crosslinked MnO_2_ hydrogel (Figure S19). Compared with A‐MnO_2_, the non‐A‐MnO_2_ electrode exhibits significantly inferior cycling stability (Figure S20). This indicates that the improved electrochemical performance of A‐MnO_2_ originates from the synergistic effect of the 3D framework and crosslinked structure, rather than from Mn vacancies alone. Specifically, although Mn vacancies can provide additional active sites and facilitate ion diffusion, the interconnected 3D porous architecture plays a crucial role in maintaining structural integrity and suppressing nanosheet restacking during repeated cycling. These results collectively demonstrate the pivotal role of the aerogel structure in enhancing the cycling stability of the MnO_2_ cathode. Specifically, the interconnected 3D porous architecture can effectively suppress the severe restacking and aggregation of MnO_2_ nanosheets during repeated charge/discharge processes, thereby maintaining sufficient electrolyte‐accessible active sites and continuous ion/electron transport pathways. The porous architecture can also facilitate electrolyte penetration and shorten ion diffusion pathways, contributing to more homogeneous electrochemical reactions throughout the electrode. In addition, the Ni^2+^‐crosslinked 3D framework can effectively alleviate the structural stress and volume variation associated with Zn^2+^ insertion/extraction, which helps maintain the structural integrity of the electrode during long‐term cycling. As a result, A‐MnO_2_ electrode can better maintain its structural stability and electrochemical activity during long‐term cycling, leading to improved cycling performance. It is worth noting that the A‐MnO_2_ cathode can achieve a high energy density of 375.3 Wh kg^−1^ at a power density of 125.5 W kg^−1^ and maintain 198.2 Wh kg^−1^ at 5500.3 W kg^−1^, outperforming previously reported Mn‐based cathode materials (Figure 3h) [28, 48, 54, 55, 56, 57, 58]. These findings highlight the effectiveness of the synergistic strategy combining Mn vacancies and metal ion crosslinking in enhancing the structural stability of pristine MnO_2_, thereby improving its electrochemical performance. To move closer to practical application scenarios, the flexible pouch cell with A‐MnO_2_ as the cathode was assembled and tested. It can be seen that the Zn//A‐MnO_2_ pouch cell can deliver a stable open‐circuit voltage of 1.4 V even in a curled state (Figure S21). Impressively, at a current density of 1.0 A g^−1^, the discharge capacity remains at 200.1 mAh g^−1^ after 100 cycles, with a capacity retention of 82.4% (Figure 3i). Simultaneously, the CE almost remains 99.1%, demonstrating good reversibility. Furthermore, the assembled pouch cell can power Light Emitting Diodes (LEDs) even under a large bending state, demonstrating the practical feasibility of this battery system in flexible energy storage devices.

### 2.4 Energy Storage Mechanism and Structural Evolution

**FIGURE 4:**
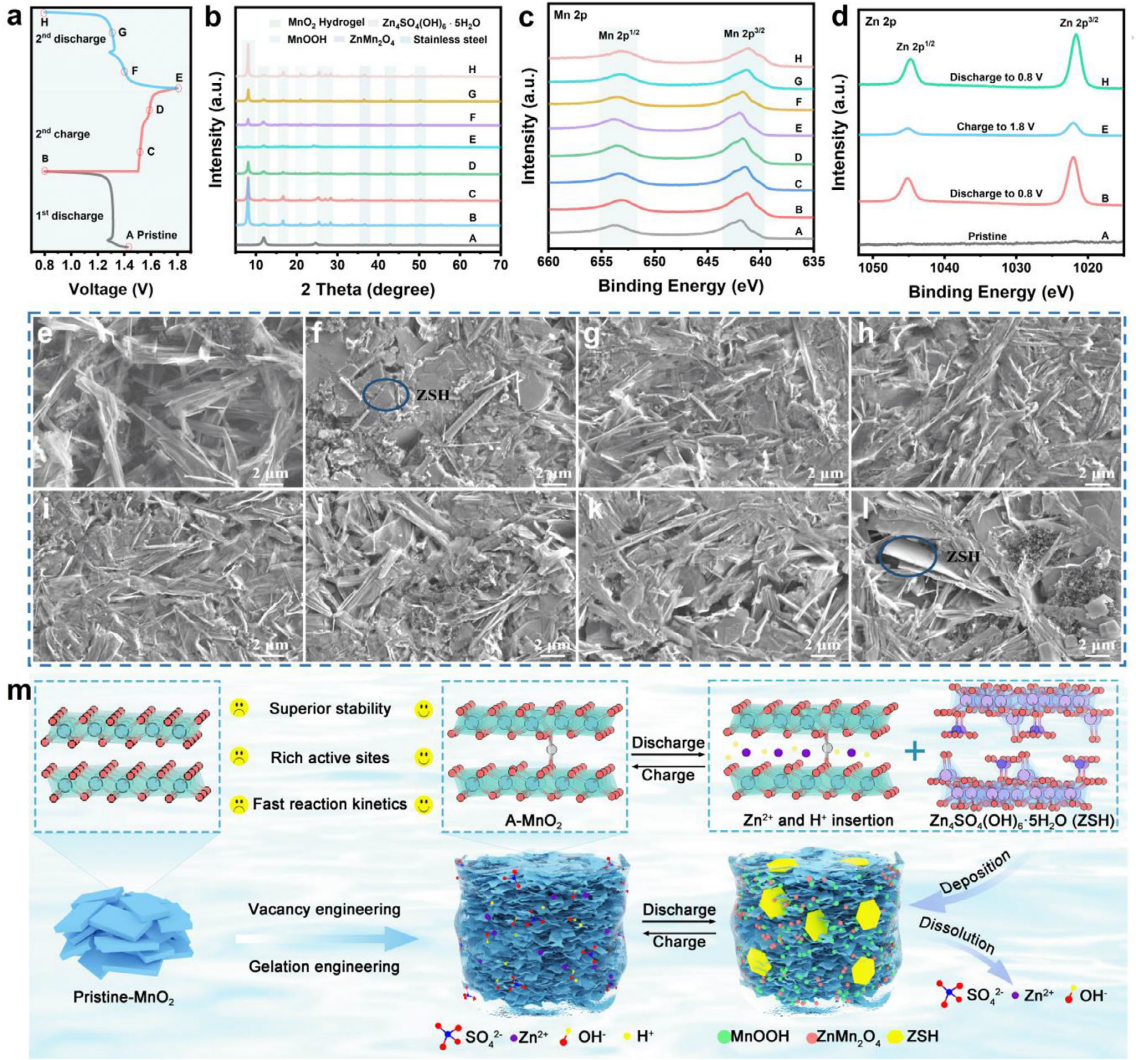
Energy storage mechanism and structural evolution of A‐MnO_2_ cathode. (a) GCD curves with selected points for ex situ characterization. (b) Ex situ XRD patterns at the selected points. (c) Mn 2p and (d) Zn 2p XPS spectra of A‐MnO_2_ collected at the selected points. (e–l) Ex situ SEM images at various states marked in (a). Figure 4e–l correspond to selected points from A to H. (m) Schematic diagram of aqueous Zn//A‐MnO_2_ battery storage mechanism.

To probe into the energy storage mechanism and structural evolution of A‐MnO_2_ cathode, ex situ XRD, XPS and SEM measurements were conducted at the selected cutoff voltages (Figure 4a). Before the GCD test, the XRD pattern of pristine A‐MnO_2_ electrode (state A) shows diffraction peaks similar to those of the powder sample, along with additional peaks originating from the stainless‐steel current collector (Figure 4b). During the first discharging process (state B), characteristic peaks of MnOOH (JCPDS No. 24–0713), ZnMn_2_O_4_ (JCPDS No. 24–1133), and Zn_4_SO_4_(OH)_6_·5H_2_O (ZSH, JCPDS No. 39–0688) are observed [52, 59, 60], confirming the co‐insertion of Zn^2+^ and H^+^. The formation of ZHS is attributed to the reaction of Zn^2+^ and SO_4_^2–^ with OH^–^ ions generated by H^+^ intercalation. These characteristic peaks gradually diminish and eventually disappear during the subsequent charging process (states C–E), and then progressively reappear in the second discharging process (states F–H), implying the reversible co‐extraction/insertion of Zn^2+^ and H^+^ accompanied by the dissolution/deposition of ZSH. Specially, the characteristic peaks of A‐MnO_2_ remain unchanged throughout the entire cycling process, indicating that metal ion crosslinking strengthens the stability of the crystal structure. Moreover, ex situ SEM images further confirm the deposition/dissolution of ZSH, as shown in Figure 4e–l. When the voltage drops to 0.8 V during the first and second cycles, the flake‐like ZSH appears on the electrode surface (Figure 4f,l). The SEM‐EDS mapping images also provide evidence for the formation of ZHS sheets (Figure S22). However, deposited ZSH sheets disappear after charging to 1.8 V (Figure 4i), which is consistent with ex situ XRD analysis. This suggests that the A‐MnO_2_ cathode maintains the structural stability throughout the reaction process, thereby enhancing its cycling performance.

Ex situ XPS analysis was performed to reveal the evolution of Mn valence states throughout the cycling process (Figure 4c). During the first discharging process (states A‐B), a pronounced shift of the Mn 2p XPS peaks toward lower binding energies is observed, reflecting a reduction in the Mn valence state associated with Zn^2+^/H^+^ insertion [61]. Subsequently, during the second charging process (states C–E), the Mn 2p XPS peaks exhibit a continuous shift toward higher binding energies, and then reversibly return after the second discharging process (states F–H), indicating the reversible change in the Mn valence states upon Zn^2+^/H^+^ insertion and extraction. In addition, the high‐resolution Zn 2p XPS spectra of A‐MnO_2_ are shown in Figure 4d. In the pristine state, no Zn 2p XPS signal is observed, indicating the absence of zinc ions [62]. When discharged to 0.8 V, strong Zn 2p signals emerge, while their intensity markedly diminishes after charging to 1.8 V, suggesting that the insertion and extraction of Zn^2+^ are reversible during the cycling process. The presence of Zn signals at the fully charged state was likely due to the surface adsorption of Zn^2^^+^ from the residual electrolyte. According to the above discussion, the A‐MnO_2_ cathode exhibits excellent reversibility and structural stability. The energy storage mechanism is attributed to the co‐insertion/extraction of H^+^ and Zn^2+^, accompanied by the reversible formation and dissolution of Zn_4_SO_4_(OH)_6_·5H_2_O flakes, as illustrated in Figure 4m. In situ Raman spectroscopy was also conducted to confirm the reversible insertion of Zn^2+^/H^+^ in the A‐ MnO_2_ cathode (Figure S23). Notably, the Raman peak at 645 cm^−1^ corresponds to the symmetric stretching vibration (ν_1_) of the Mn─O bond in MnO_6_ octahedra, which is closely associated with Zn^2+^ insertion/extraction. During the initial discharge of the Zn//A‐MnO_2_ batteries from the open‐circuit voltage (≈1.42 V) to 0.8 V, the ν_1_ band exhibits a redshift from 642.3 to 633.3 cm^−1^, which can be attributed to the insertion of Zn^2+^/H^+^ and the accompanying distortion of MnO_6_ octahedra. During the subsequent charge/discharge process, the Raman shift of the ν_1_ band exhibits a reversible trend, indicating enhanced structural stability during the insertion/extraction of Zn^2+^ and H^+^.

### 2.5 DFT Calculation Results and Kinetics Analysis

**FIGURE 5:**
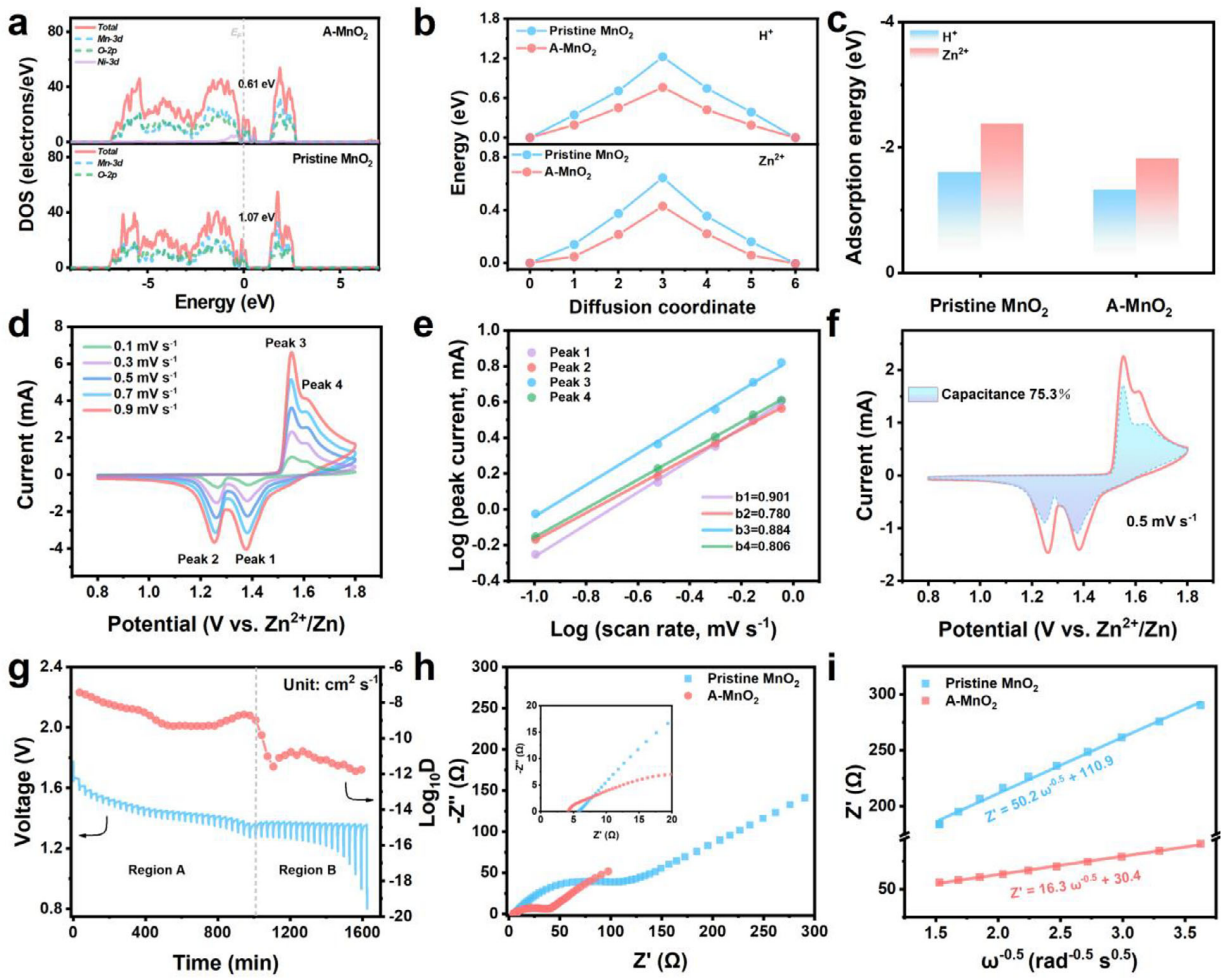
The DFT calculation results and kinetics analysis of A‐MnO_2_ cathodes. (a) TDOS of pristine MnO_2_ and A‐MnO_2_. (b) Migration energy barriers of H^+^ and Zn^2+^ in pristine MnO_2_ and A‐MnO_2_. (c) Adsorption energies of H^+^ and Zn^2+^ on pristine MnO_2_ and A‐MnO_2_. (d) CV curves of A‐MnO_2_ at different scan rates. (e) Log (peak current) vs. log (scan rate) plots of four peaks at (d). (f) The capacitive contribution of A‐MnO_2_ at a scan rate of 0.5 mV s^−1^. (g) GITT profiles and the corresponding ion diffusion coefficient of A‐MnO_2_. (h) Nyquist plots. (i) Linear plots of Z′ vs. ω^−0.5^ at the low‐frequency region.

DFT calculations and experimental analysis were utilized to understand the electronic properties and electrochemical kinetic processes of pristine MnO_2_ and A‐MnO_2_ (Figures S24a and S25a). The total density of states (TDOS) of pristine MnO_2_ and A‐MnO_2_ are shown in Figure 5a. A‐MnO_2_ displays a narrower bandgap energy of 0.61 eV compared to pristine MnO_2_ (1.07 eV), which confirms its improved electronic conductivity, suggesting that the introduction of Mn vacancies and metal ions has a positive effect on the TDOS of pristine MnO_2_. Moreover, the Zn^2+^/H^+^ diffusion pathways and the corresponding diffusion barriers in pristine MnO_2_ and A‐MnO_2_ structure are calculated and displayed in Figures S24b, S25b, and S5b. With metal ion crosslinking, the diffusion barriers of Zn^2+^/H^+^ in A‐MnO_2_ are 0.76/0.43 eV, lower than that in pristine MnO_2_ (1.23/0.65 eV), indicating a faster Zn^2+^/H^+^ migration in A‐MnO_2_ [63]. The reduction in the diffusion barriers of Zn^2+^ and H^+^ is related to the changes in the adsorption energy between A‐MnO_2_ and these two ions [64]. As depicted in Figure 5c. the adsorption energies of Zn^2+^ and H^+^ in A‐MnO_2_ are −1.81 and −1.33 eV, while the adsorption energies of Zn^2+^ and H^+^ in pristine MnO_2_ are −2.38 and −1.61 eV. The smaller adsorption energies in A‐MnO_2_ indicate that the addition of metal ions into pristine MnO_2_ can effectively weaken the interaction between ions (Zn^2+^/H^+^) and the MnO_2_ lattice, promoting the rapid transport of ions between the MnO_2_ layers.

To further elucidate the impact of metal ion crosslinking and Mn vacancies on the charge storage kinetics, cyclic voltammetry (CV) tests at different scan speeds (0.1–0.9 mV s^−1^) are employed and described in Figure 5d and Figure S16. It can be discovered that the CV curves of the pristine MnO_2_ and A‐MnO_2_ cathodes exhibit two pairs of redox peaks and splendid reversibility. Then, the relationship between the peak current (*i*) and the scan rate (*v*) follows the formula below: *i* = *a v^b^* (*a* and *b* are variable parameters). The *b* value is a key parameter to determine the dominant modus of charge storage, in which *b* = 0.5 and *b* = 1 indicate representative diffusion‐controlled and capacitive‐controlled behaviors, respectively [65]. Through the linear fitting of log *i* and log *v* (Figure 5e), the *b* values of A‐MnO_2_ cathodes are 0.901 (Peak 1), 0.780 (Peak 2), 0.884 (Peak 3), and 0.806 (Peak 4), which are larger than those of pristine MnO_2_ cathode (Figure S26), demonstrating more capacitive‐controlled behaviors. Furthermore, the ratio of the capacitive contribution is obtained from the following equation: *i*(V)  =  *k*_1_ν  +  *k*_2_ν^1/2^, where *k*_1_ν and *k*_2_ν^1/2^ represent the capacitive and diffusion‐controlled parts, respectively [66]. Obviously, as shown in Figure 5f and Figure S27, the capacitive contribution ratios of A‐MnO_2_ (51.1%, 66.9%, 75.3%, 80.1%, and 84.5%) are higher than those of pristine MnO_2_ (36.8%, 53.7%, 69.1%, 74.4%, and 77.2%) at different scan rates (0.1, 0.3, 0.5, 0.7, and 0.9 mV s^−1^), revealing faster kinetic behaviors of A‐MnO_2_ during the energy storage process. Subsequently, galvanostatic intermittent titration technique (GITT) tests were conducted to comprehend the kinetic performance. As manifested in Figure 5g, the A‐MnO_2_ cathode presents two voltage platforms (Region A and Region B), corresponding to the intercalation of H^+^ and Zn^2+^ ions, respectively. The ion‐diffusion coefficient (*D_ion_*) of A‐MnO_2_ is between 10^−7^ and 10^−12^ cm^2^ s^−1^, higher than that of pristine MnO_2_ (10^−8^–10^−13^ cm^2^ s^−1^) (Figure S28, details described in the Supporting Information) [67]. This indicates that abundant Mn vacancies and 3D porous structures are beneficial to enhance the ion transport capability of pristine MnO_2_. Notably, the *D_ion_* of A‐MnO_2_ in Region A is clearly better than that in Region B, revealing a faster ion diffusion and lower energy barrier, in accordance with the above theoretical calculation results. In addition, the kinetic properties are further investigated using the electrochemical impedance spectra (EIS). As shown in Figure 5h, the two Nyquist plots consist of near semicircles in the high‐frequency region and oblique straight lines in the low‐frequency region. The A‐MnO_2_ cathode presents a lower ohmic resistance (4.1 Ω) than the pristine MnO_2_ cathode (5.7 Ω). Moreover, the charge transfer resistance (Rct) of A‐MnO_2_ (43.5 Ω) is significantly lower than that of pristine MnO_2_ (112.7 Ω). These results strongly demonstrate that the abundant Mn vacancies and 3D porous structure facilitate charge transfer by promoting faster ion diffusion in A‐MnO_2_. Moreover, the *D_ion_* can also be obtained based on the correlation between real part of impedance and the low‐frequency of EIS [68]. The details are shown in the Supporting Information. It can be calculated that the *D_ion_* of A‐MnO_2_ (1.6 × 10^−12^ cm^2^ s^−1^) is greater than that of pristine MnO_2_ (1.7 × 10^−13^ cm^2^ s^−1^) (Figure 5i), consistent with GITT results. On the whole, these findings demonstrate improved electrical conductivity and enhanced diffusion kinetics, thereby ensuring excellent rate performance.

## 3 Conclusion

In conclusion, we have designed Mn‐deficient aerogels by inducing the gelation of MnO_2_ nanosheets in solution using Ni^2+^ ions as crosslinking agents, thereby inhibiting nanosheet restacking and enhancing surface utilization. Specifically, the Ni^2+^‐mediated 3D framework helps alleviate the structural stress and volume fluctuations associated with repeated Zn^2+^ insertion/extraction, thereby maintaining the structural integrity of the aerogel during long‐term cycling. In addition, the strengthened intersheet interactions induced by Ni^2+^ crosslinking improve the mechanical stability of the porous network and mitigate structural collapse or pulverization during electrochemical reactions. Consequently, Ni^2+^ crosslinking not only facilitates aerogel formation but also contributes significantly to the cycling stability of the A‐MnO_2_ electrode. Benefiting from abundant Mn vacancies and 3D porous structures, the A‐MnO_2_ cathode exhibits improved conductivity, fast reaction kinetics, and superior structural stability, as evidenced by its reduced bandgap, increased ion diffusion coefficients, and uniform electrode morphology after cycling. The assembled Zn//A‐MnO_2_ batteries achieve a high specific capacity of 308.3 mAh g^−1^ at 0.1 A g^−1^, superior rate performance of 146.3 mAh g^−1^ at 5 A g^−1^, and good cycling stability with 90.6% capacity retention after 5000 cycles. Furthermore, the assembled soft‐packaged Zn//A‐MnO_2_ batteries deliver a high specific capacity of 242.9 mAh g^−1^ and remarkable capacity retention of 82.4% after 100 cycles at 1 A g^−1^. This work highlights the critical role of cation‐vacancy engineering in manganese‐based cathodes and provides new insights into the rational design and fabrication of materials assembled from 2D nanosheets. Moreover, the ion‐induced gelation strategy is readily scalable under mild aqueous conditions and may provide a general route for constructing 3D architectures of other transition metal oxides with suitable surface functional groups.

## Author Contributions

**Xinyu Huang**: visualization, validation, software. **Feng Yang**: resources, writing – review and editing, supervision. **Caichao Ye**: software, methodology, formal analysis. **Bowen Li**: methodology, investigation, data curation. **Shulong Chang**: validation, visualization, software. **Yalei Wang**: writing – review and editing, data curation, methodology, conceptualization, formal analysis. **Yahui Xue**: supervision, resources, funding acquisition, writing – review and editing, project administration.

## Conflicts of Interest

The authors declare no conflicts of interest.

## Supporting information




**Supporting File**: advs76281‐sup‐0001‐SuppMat.docx.

## Data Availability

The data that support the findings of this study are available from the corresponding author upon reasonable request.
