# Supplementary material for: Metal Ion‐Induced Fast Gelation of 2D MnO2 Nanosheets With Cationic Vacancies for Durable Aqueous Zinc‐Ion Batteries
Source: Adv Sci (Weinh). 2026 Jun 25:e76281. Online ahead of print. doi: 10.1002/advs.76281 (PMC13336370; doi:10.1002/advs.76281)
Supplement: Supplementary file 1 — Supporting File: advs76281‐sup‐0001‐SuppMat.docx. [file ADVS-9999-e76281-s001.docx]

Supporting Information

Metal Ion-Induced Fast Gelation of 2D MnO_2_ Nanosheets with Cationic Vacancies for Durable Aqueous Zinc-Ion Batteries

Yalei Wang, Xinyu Huang, Shulong Chang, Bowen Li, Caichao Ye*, Feng Yang, Yahui Xue*

**Experimental sections**

***Preparation of the MnO_2_ nanosheets***

The MnO_2_ nanosheets (pristine MnO_2_) were prepared by a top-down delamination strategy. K-birnessite crystals were obtained via a hydrothermal method. Briefly, 3 mmol of KMnO_4_ and 1.8 mol of KOH were dissolved in 84 mL of H_2_O. Then, 8 mL of H_2_O containing 3 mmol of MnCl_2_·4H_2_O was added immediately. After cooling to room temperature, the resulting mixture was transferred into a Teflon-lined stainless steel autoclave and maintained at 175 °C for 2 days. The resultant product, K-birnessite crystals, was successively treated with 250 mL of 0.5 M (NH_4_)_2_S_2_O_8_ at 60 °C for 12 h and 1 M HCl at room temperature for 2 h. Then, the protonated H-birnessite was immersed in 50 mL of tetramethylammonium hydroxide (TMAOH) aqueous solution with mechanical shaking for 2 days. The as-obtained suspension was repeatedly washed with deionized water until the supernatant became neutral. Finally, the sediment was redispersed in deionized water and delaminated by shaking at 120 rpm for 2 days.

***Assembly of A-MnO_2_***

0.2 mL of 1 M NiCl_2_ solution was added into 5 mL MnO_2_ suspension (10 mg mL^−1^) and the gel was formed in a few seconds (NiCl_2_/MnO_2_ mass ratio = 1:2). The formed hydrogel was pre-treated with liquid nitrogen and then freeze-dried to obtain the aerogel (A-MnO_2_). The obtained sample was directly dried in a vacuum drying oven and denoted as non-A-MnO_2_. Moreover, hydrogels crosslinked by other metal ions were obtained using the same procedure by replacing NiCl_2_ with KCl or AlCl_3_. By adjusting the NiCl_2_ concentration to 1.2 M and 1.6 M, comparison samples with NiCl_2_/MnO_2_ mass ratios of 3:5 and 4:5 were also prepared.

***Characterizations***

The crystal structures of samples were obtained through X-ray diffraction (XRD, Rigaku, SmartLab). The morphology of samples was characterized by the scanning electron microscope (SEM, Zeiss, Merlin) and transmission electron microscope (TEM, FEI, Tecnai F30) with energy dispersive spectroscopy (EDS) for elemental analysis. The high-angle annular dark-field scanning transmission electron microscopy (HAADF-STEM) images and electron energy-loss spectroscopy (EELS) data were collected by a Titan Themis G2 instrument. The atomic force microscope (AFM) images were taken on a Cypher ES atomic force microscope. X-ray photoelectron spectroscopy (XPS) was carried out by a Thermo Scientific ESCALAB 250Xi spectrometer. The specific surface area and pore size distribution were examined by the surface area analyzer (Micromeritics, ASAP 2460). Electron paramagnetic resonance (EPR, Bruker, EMX Plus) were carried out to explore the vacancies in the samples. Raman spectra were recorded using a HORIBA XploRA PLUS spectrometer. The Zeta potential was measured by a Malvern Zetasizer Nano ZS analyzer. The contact angle between electrodes and electrolytes was measured using a Dataphysics OCA25 contact angle instrument. Fourier transform infrared (FTIR) spectra were collected using a Nicolet iS50 spectrometer.

***Electrochemical Measurements***

The working electrode was prepared by mixing the active material (A-MnO_2_ or pristine MnO_2_), conductive agent (carbon black), and binder (polyvinylidene fluoride) at a mass ratio of 7:2:1 in the solvent N-methyl-2-pyrrolidone. The slurry was evenly ground and coated on the steel mesh, then it was dried under vacuum at 60 °C for 12 h. The mass loading of the active materials was approximately 1.2 mg cm^−2^. The coin cells were assembled using A-MnO_2_ or pristine MnO_2_ as the cathode, Zn foil as the anode, glass fiber membrane as the separator, and 2 M ZnSO_4_/0.1 M MnSO_4_ mixed solution as the electrolyte, respectively. In contrast, Zn//A-MnO_2_ batteries batteries using an MnSO_4_-free electrolyte were also assembled. Battery performance tests and Galvanostatic Intermittent Titration Technique (GITT) were carried out using LAND battery testing systems. Cyclic voltammetry (CV) and electrochemical impedance spectra (EIS) measurements were conducted on a CHI760E electrochemical workstation.

The ion diffusion coefficient (*D_ion_*) can be calculated from GITT results using the following equation:

$$\text{D}_{\text{ion}}\text{=}\frac{\text{4}\text{L}^{\text{2}}}{\text{πτ}}\left( \frac{\text{∆}\text{E}_{\text{s}}}{\text{∆}\text{E}_{\text{τ}}} \right)^{\text{2}}$$

where *τ* and *L* stand for the constant current pulse time and ion diffusion length, respectively. Δ*E_s_* represents the voltage difference between neighboring equilibrium states. ∆*E_τ_* corresponds to the voltage variation caused by the galvanostatic charge/discharge.

The ion diffusion coefficient (*D_ion_*) can also be calculated from EIS results as follows:

$$Z^{\text{'}}=R_{s}+{R_{f}+R}_{ct}+\sigma_{w}\omega^{-0.5}$$

$$\text{D}_{\text{ion}}\text{=}\frac{\text{R}^{\text{2}}\text{T}^{\text{2}}}{\text{2}\text{A}^{\text{2}}\text{n}^{\text{4}}\text{F}^{\text{4}}\text{C}^{\text{2}}\text{σ}_{\text{w}}^{\text{2}}}$$

where *R, T, A, n, F,* and *C* refer to the gas constant (8.314 J mol^−1^∙K^−1^), absolute temperature (298 K), electrode area, transferred electron number, Faraday constant (96,500 C mol^−1^), and ion concentration, respectively. $\sigma_{w}$ is the slope obtained from the linear fit of the relationship between the low-frequency region and the real part of the impedance.

***Theoretical Calculations***

Density functional theory (DFT) calculations were performed using the Vienna Ab-initio Simulation Package (VASP) with the projector augmented wave (PAW) method.^[1]^ The gradient-corrected exchange correlation functional was treated by the generalized gradient approximation (GGA) with the Perdew-Burke-Ernzerhof (PBE) functional.^[2]^ The computational model was constructed based on a two-layered system with an interlayer spacing of 7.0 Å. A plane-wave basis with an energy cutoff of 450 eV was used, and the geometry relaxation was carried out until the forces on each atom were below 0.02 eV/Å. Self-consistent calculations were conducted with an energy convergence threshold of 10^−5^ eV. The Brillouin zones were sampled using Γ-centered Monkhorst-Pack k-point meshes of 3 × 4 × 1 for the models. The DFT-D3 method considering van der Waals interaction was adopted for the adsorption system.^[3]^ The climbing image-nudged elastic band (CI-NEB) method was used to study migration pathways and corresponding energy barriers.^[4]^

**Supporting Figures and Discussions**


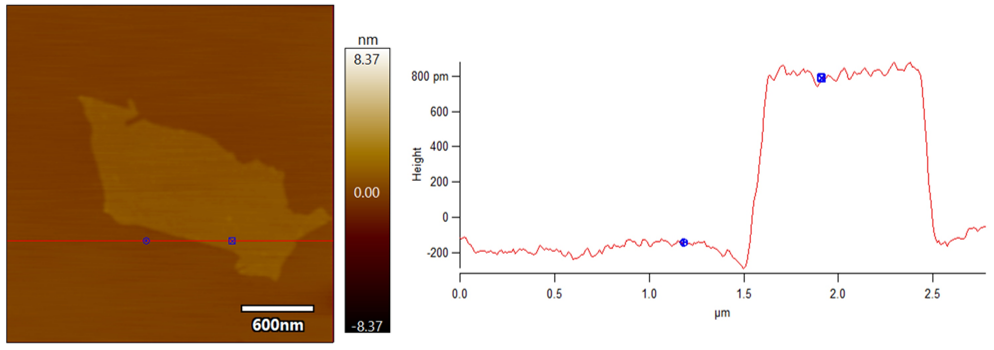
\

**Figure S1**. AFM and height images of pristine MnO_2_ nanosheets.


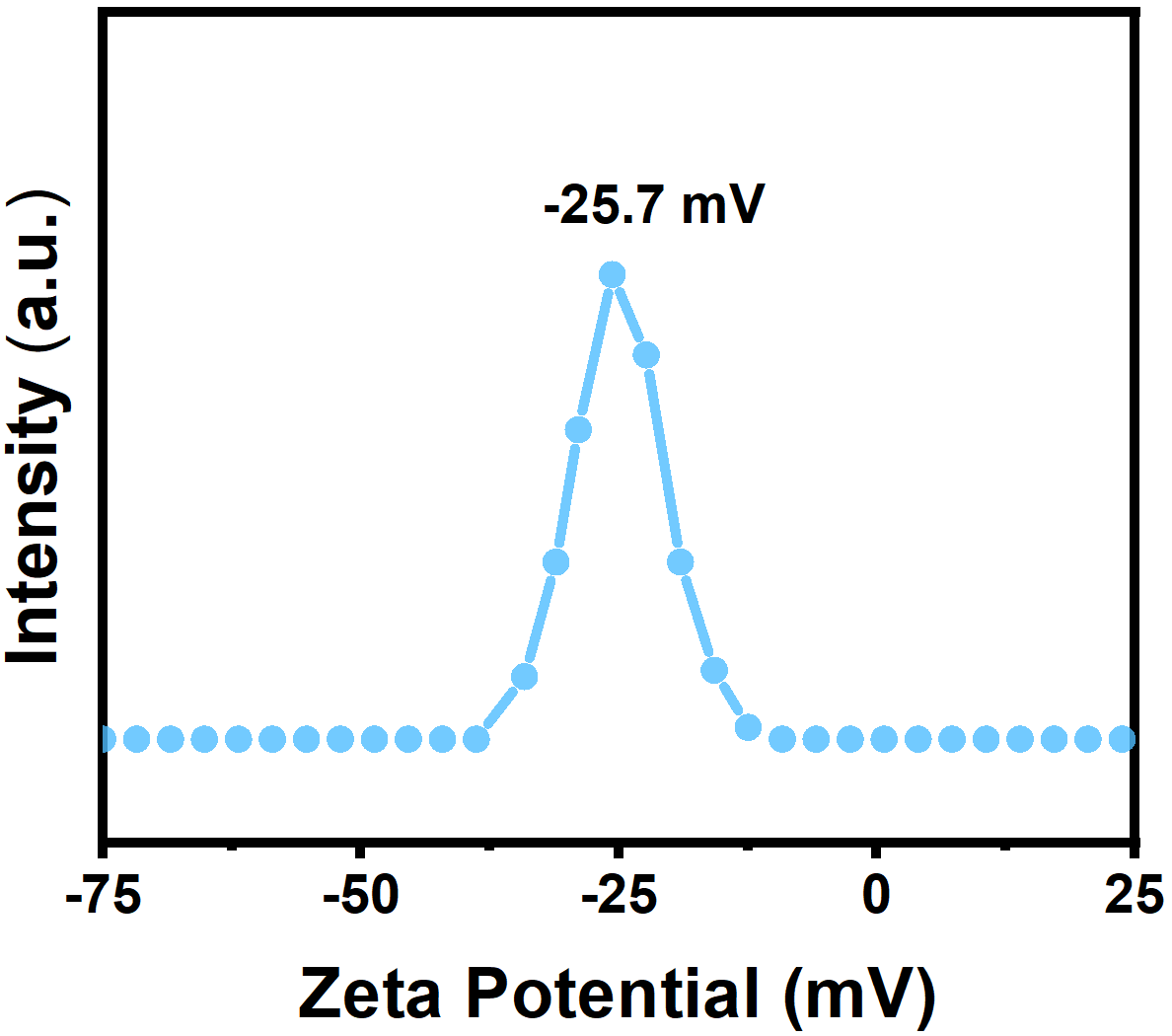


**Figure S2**. Zeta potential of pristine MnO_2_ solution.


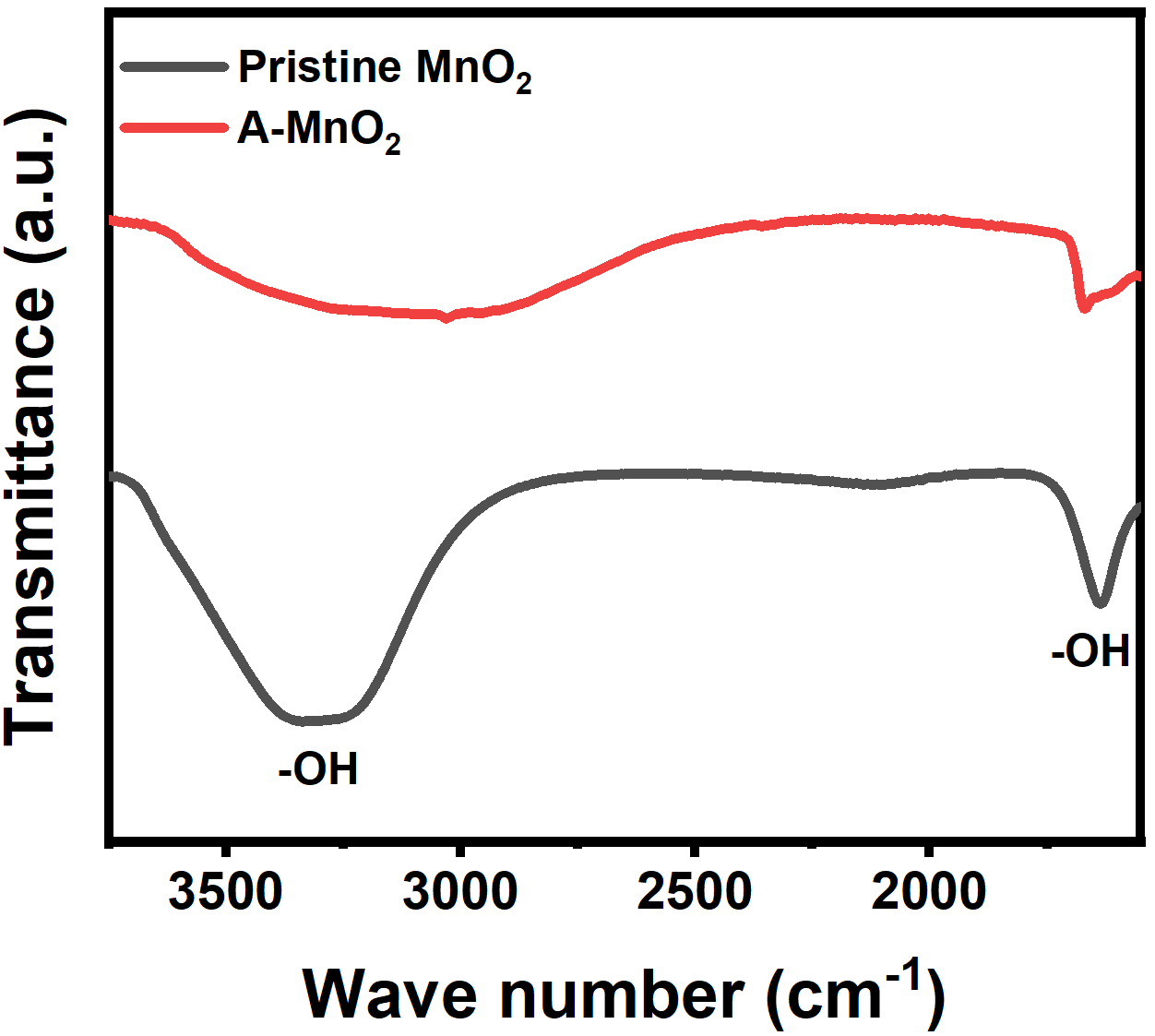


**Figure S3**. FTIR spectra of pristine MnO_2_ and A-MnO_2_.


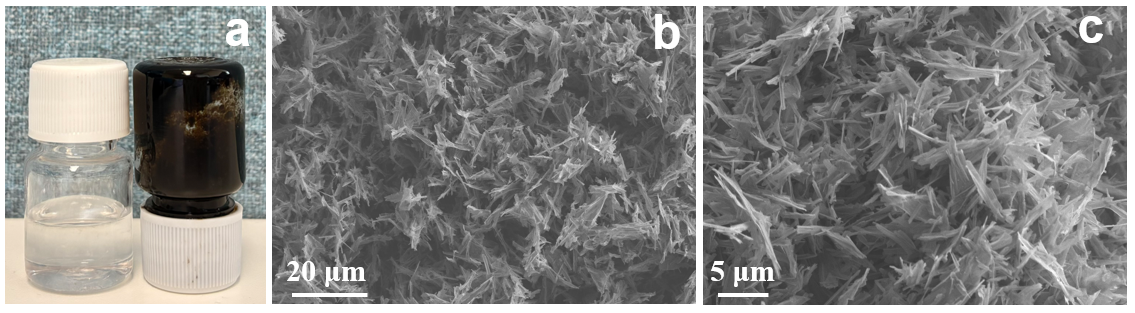


**Figure S4**. Photos and SEM images of freeze-dried MnO_2_ hydrogels initiated with K^+^ (a-c).


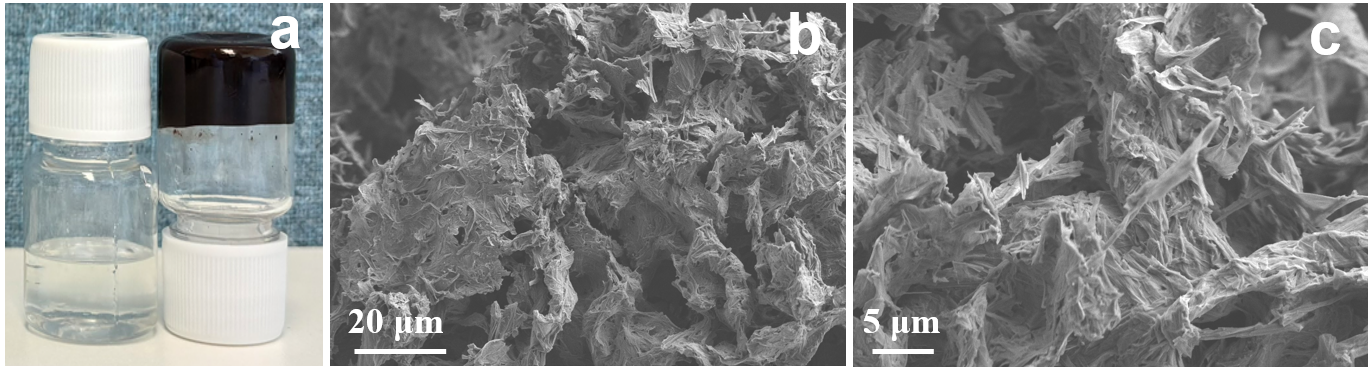


**Figure S5**. Photos and SEM images of freeze-dried MnO_2_ hydrogels initiated with Al^3+^ (a-c).


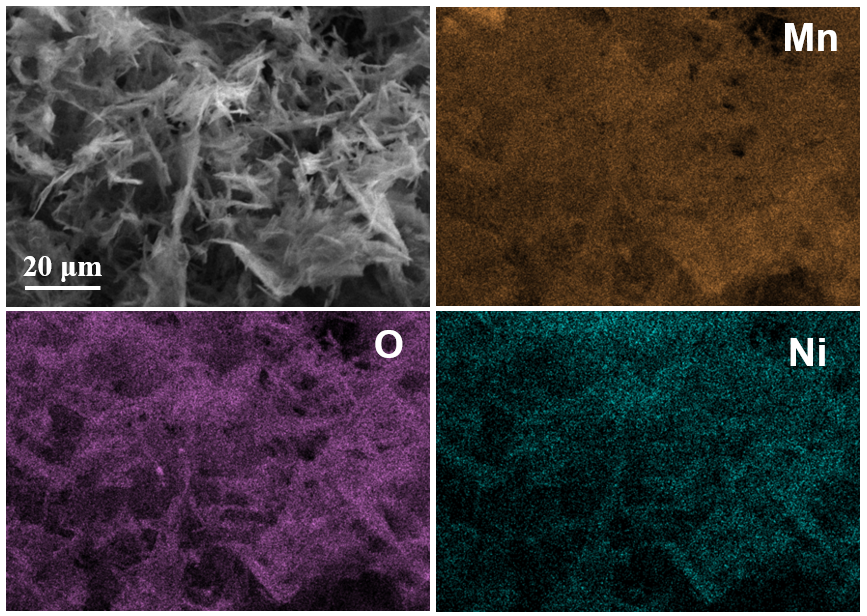


**Figure S6**. SEM and the corresponding elemental mapping images of A-MnO_2_.


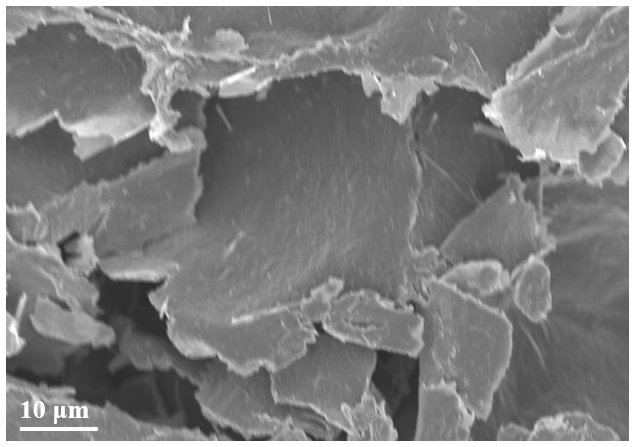


**Figure S7**. SEM images of pristine MnO_2_.


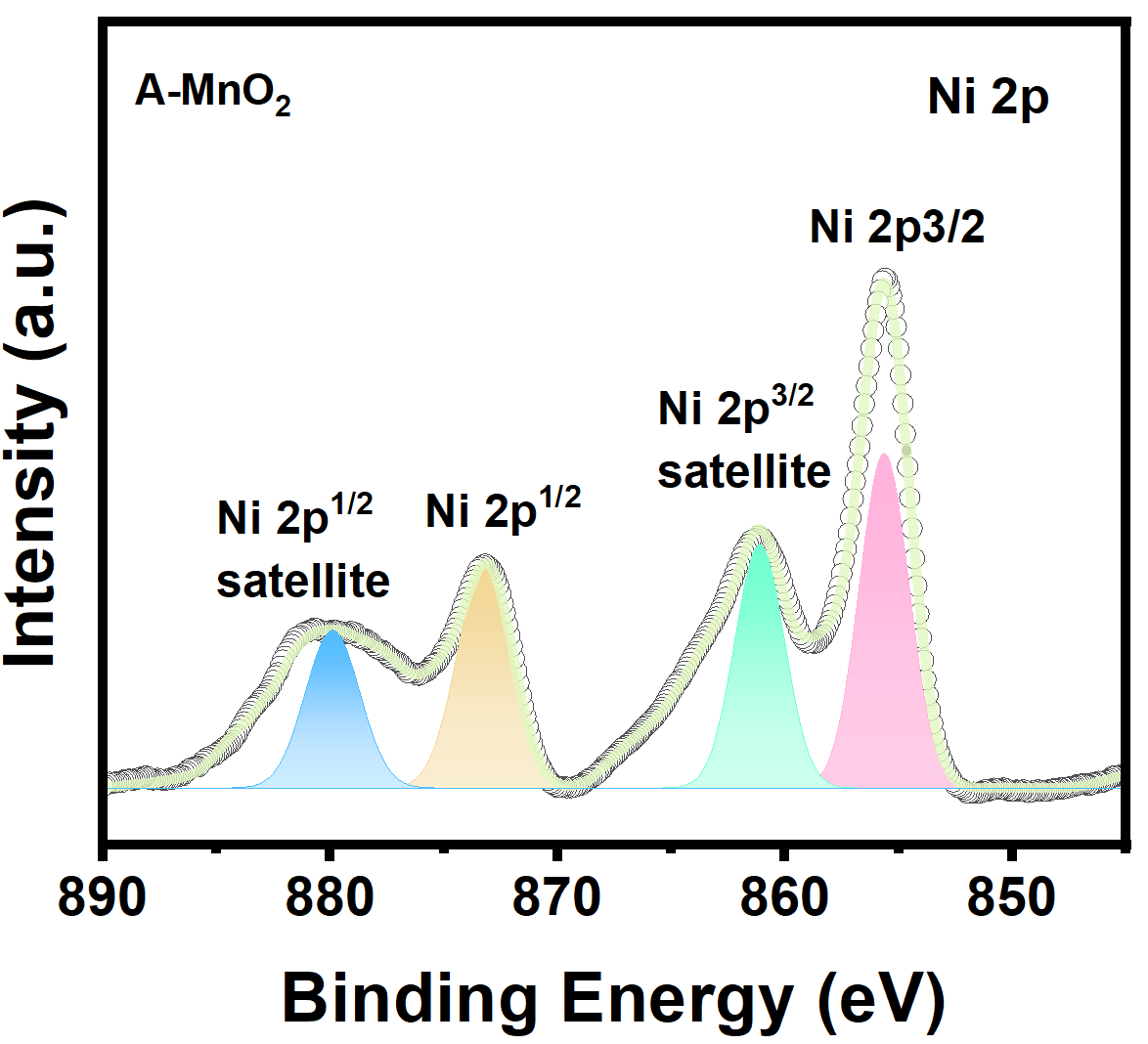


**Figure S8**. XPS spectra of Ni 2p.


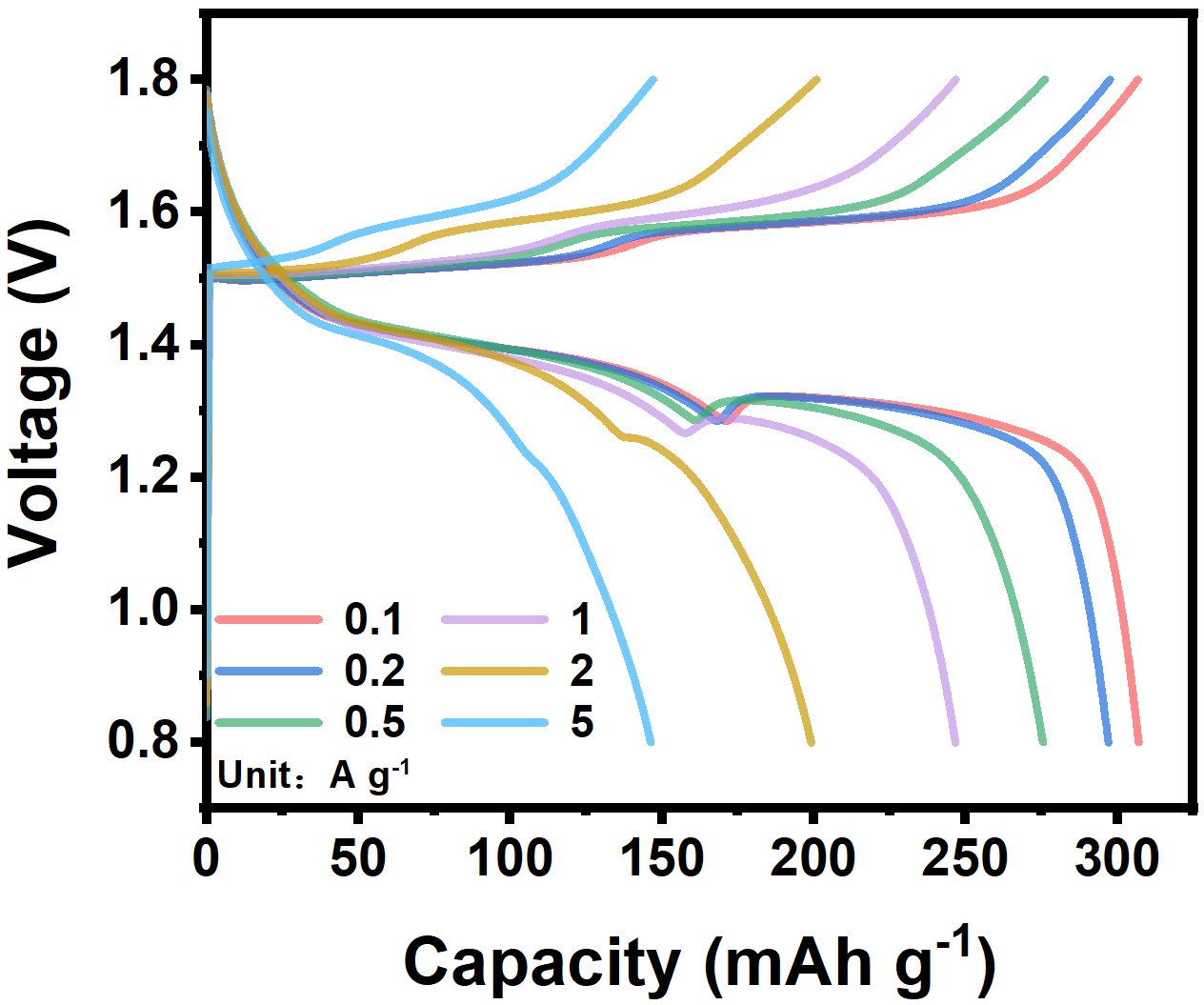


**Figure S9**. Galvanostatic charge/discharge profiles of A-MnO_2_ at different current densities.


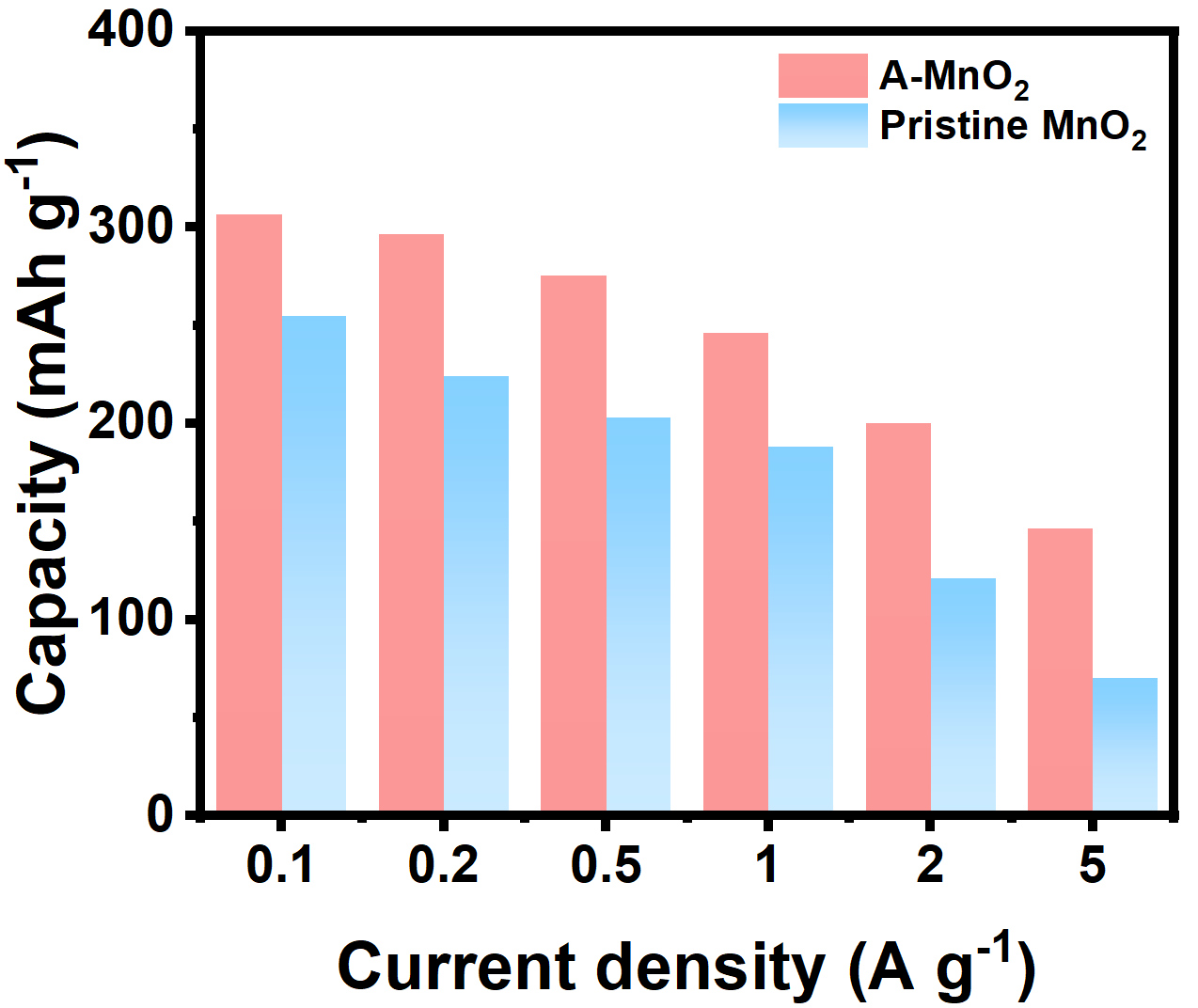


**Figure S10**. The average capacities of pristine MnO_2_ and A-MnO_2_.


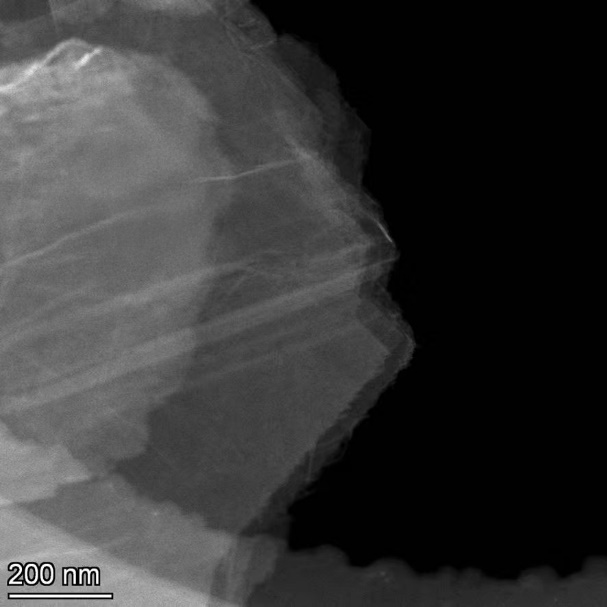


**Figure S11**. The TEM image of A-MnO_2_ after 100 cycles at 0.1 A g^−1^.


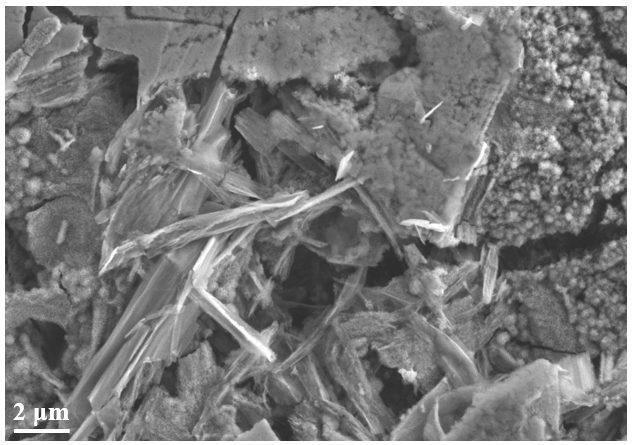


**Figure S12**. The SEM image of A-MnO_2_ cathodes after 5000 cycles at a current density of 5 A g^−1^.


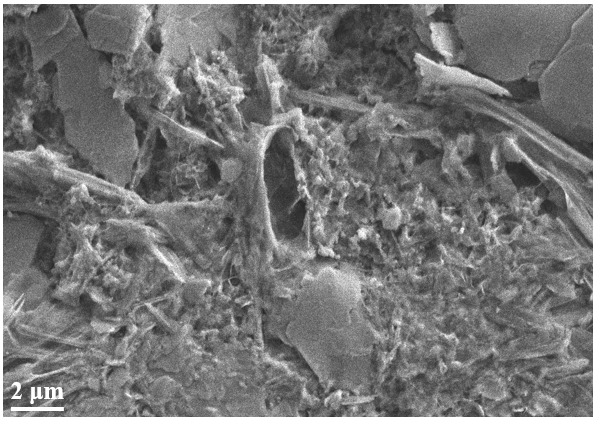


**Figure S13**. The SEM image of pristine MnO_2_ cathodes after 2000 cycles at a current density of 5 A g^−1^.


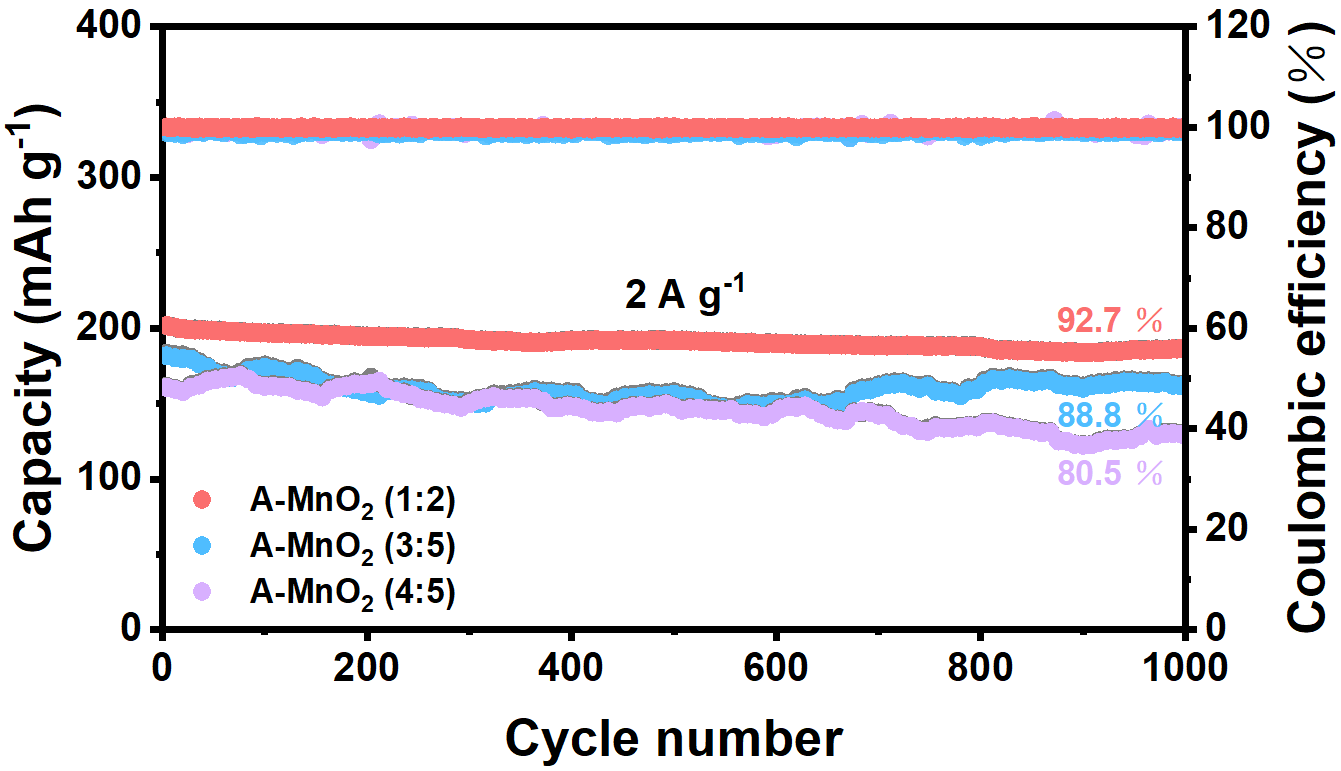


**Figure S14**. Long-term cycling performance of A-MnO_2_ (1:2), A-MnO_2_ (3:5), and A-MnO_2_ (4:5) at 2 A g^−1^.


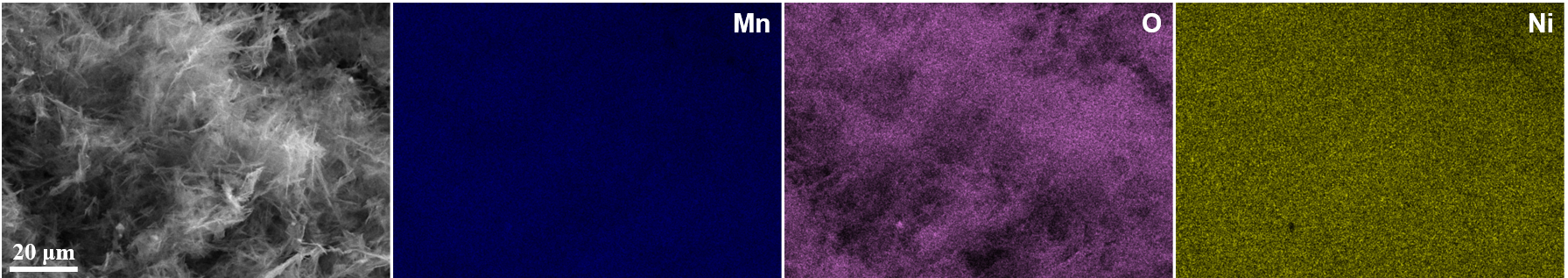


**Figure S15**. SEM and the corresponding elemental mapping images of A-MnO_2_ (1:2) after 1000 cycles at 2 A g^−1^.


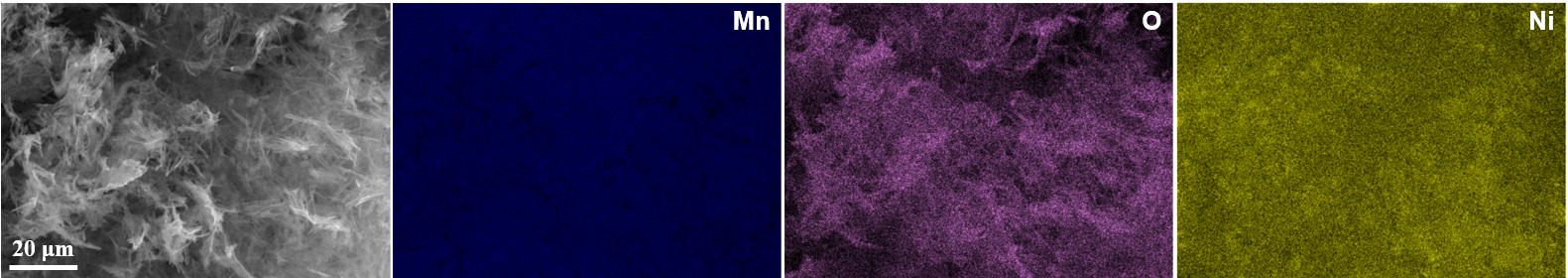


**Figure S16**. SEM and the corresponding elemental mapping images of A-MnO_2_ (3:5) after 1000 cycles at 2 A g^−1^.


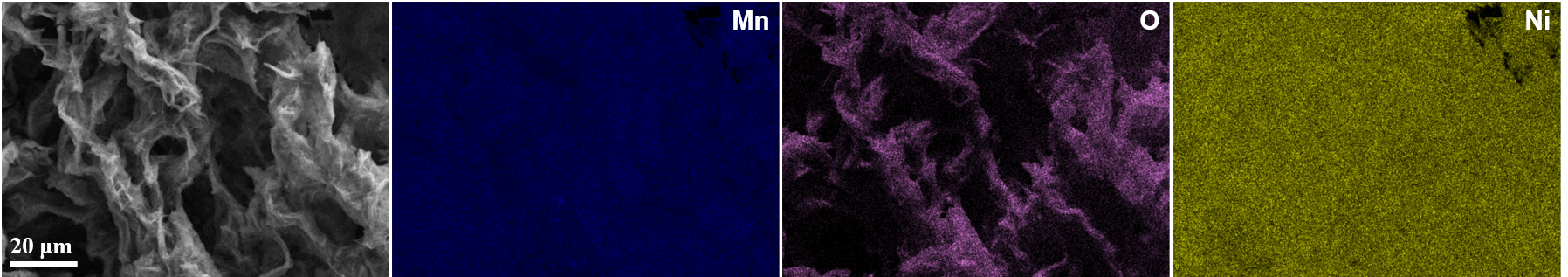


**Figure S17**. SEM and the corresponding elemental mapping images of A-MnO_2_ (4:5) after 1000 cycles at 2 A g^−1^.


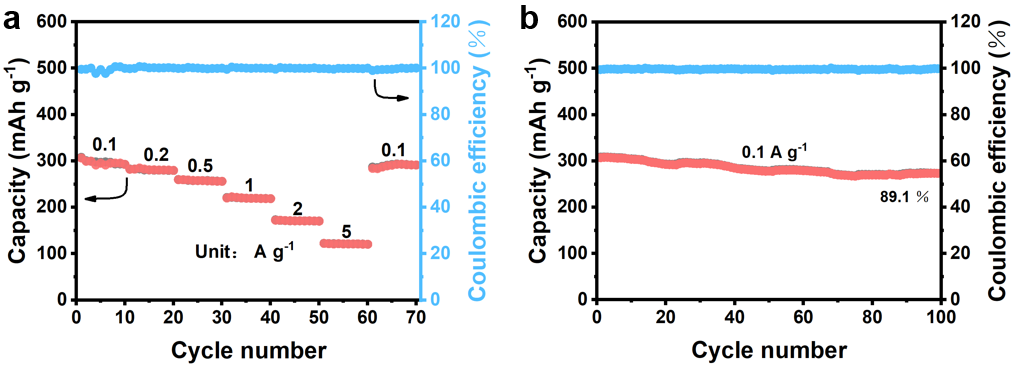


**Figure S18**. Rate capability (a) and cycling performance (b) of A-MnO_2_ electrodes without MnSO_4_ additive.


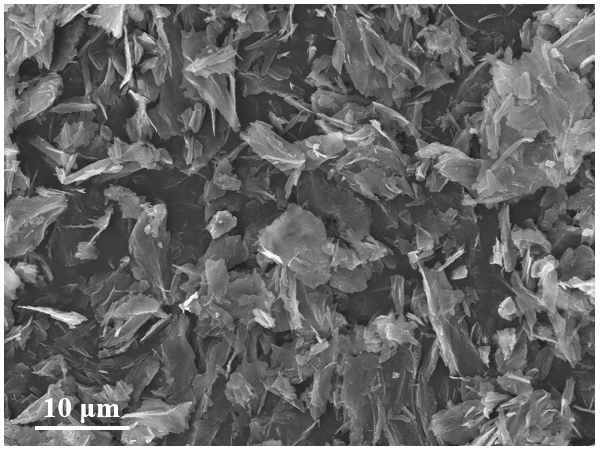


**Figure S19**. SEM images of non-A-MnO_2_.


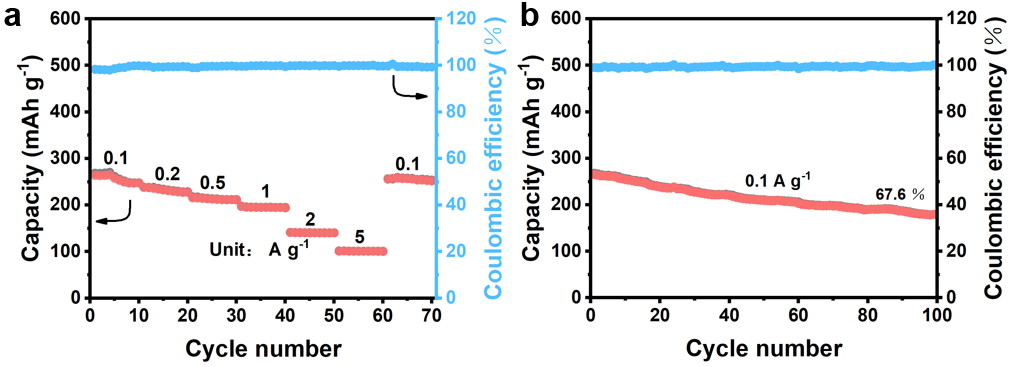


**Figure S20**. Rate capability (a) and cycling performance (b) of non-A-MnO_2_ electrodes.


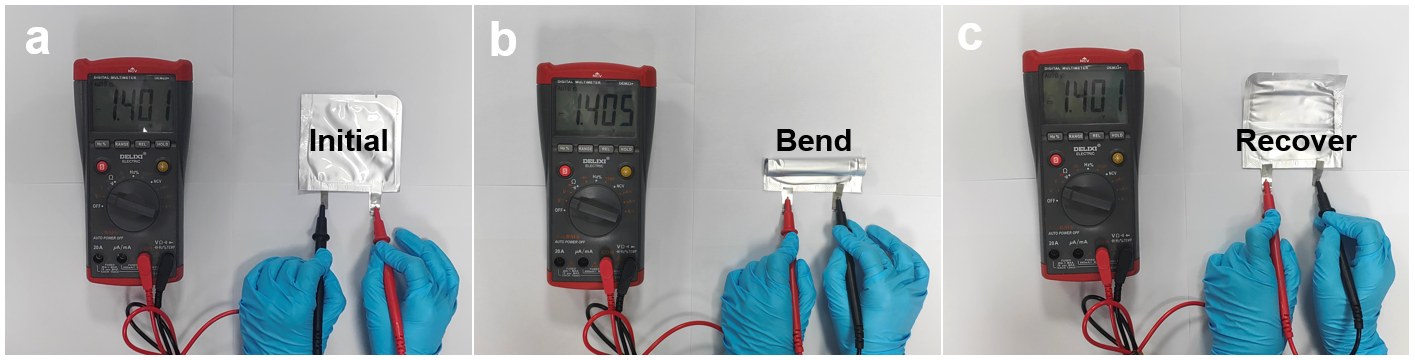


**Figure S21**. The open circuit voltage of the soft-packaged battery at various mechanical deformation states.


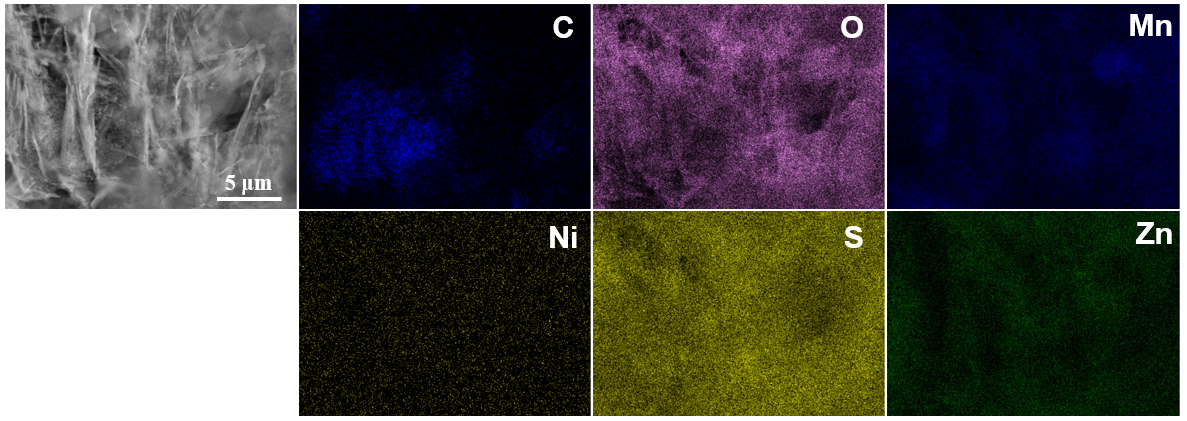


**Figure S22**. SEM and the corresponding elemental mapping images of A-MnO_2_ cathodes when discharged to point H.


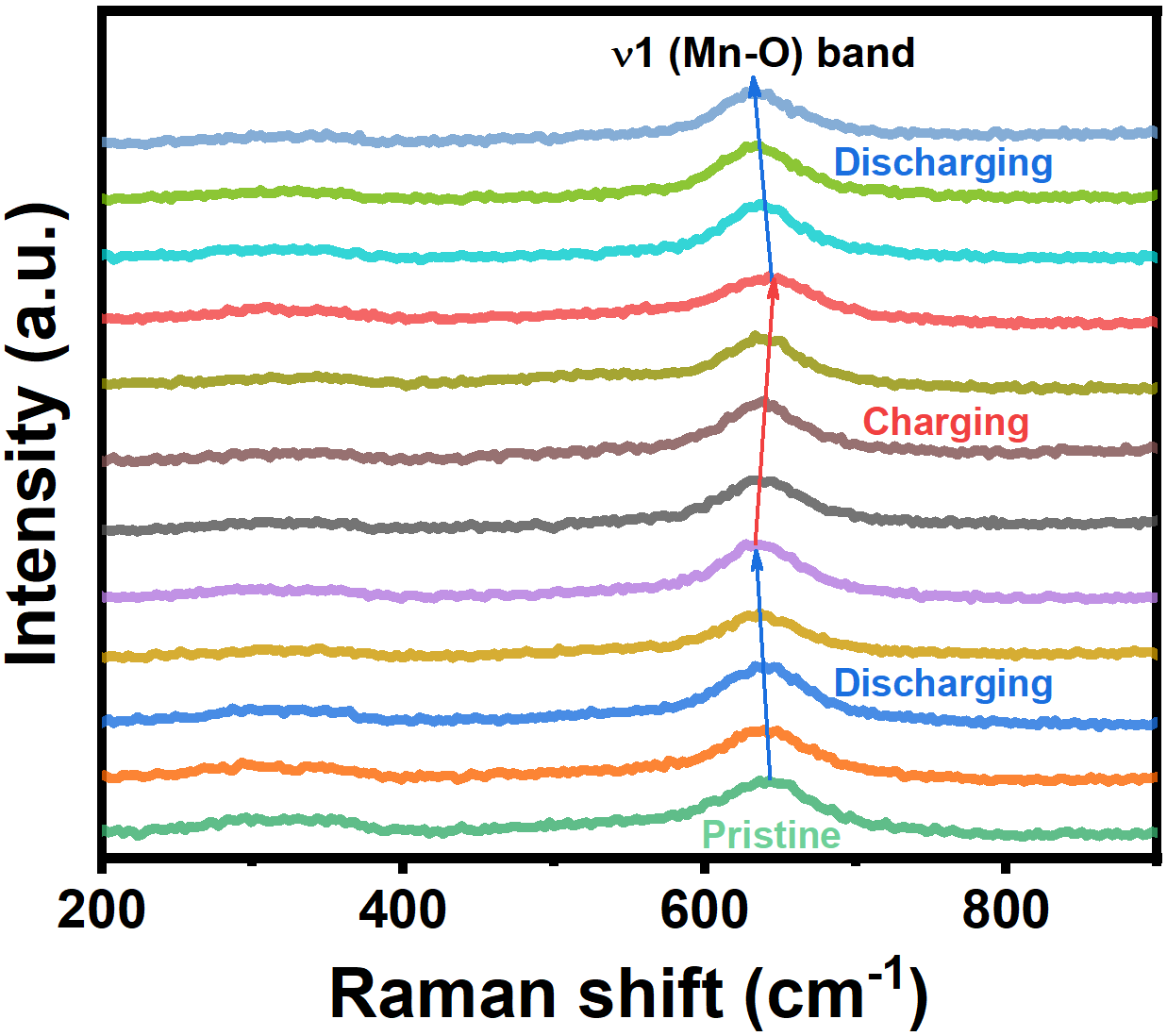


**Figure S23**. In situ Raman spectra of the A-MnO_2_ cathode during different charge/discharge processes.


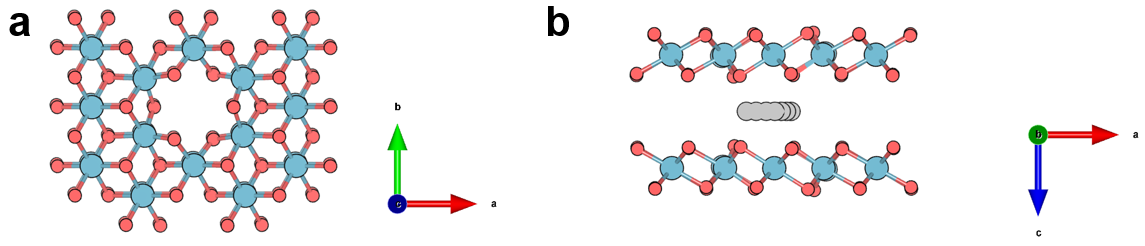


**Figure S24.** a) Structure diagrams of pristine MnO_2_. b) Schematic diagram of Zn^2+^/H^+^ migration paths in pristine MnO_2_.


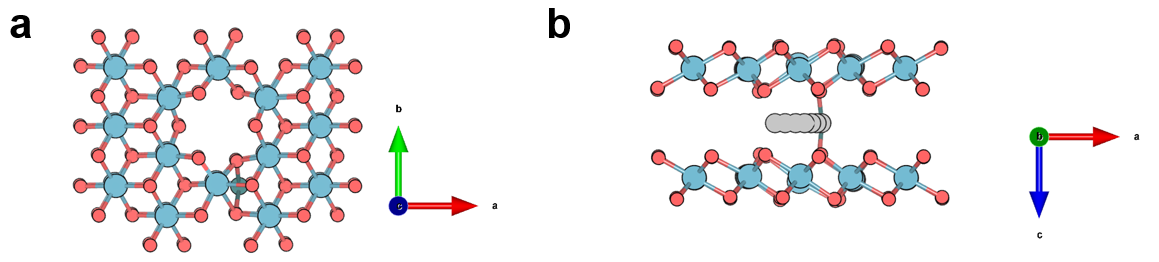


**Figure S25.** a) Structure diagrams of A-MnO_2_. b) Schematic diagram of Zn^2+^/H^+^ migration paths in A-MnO_2_.


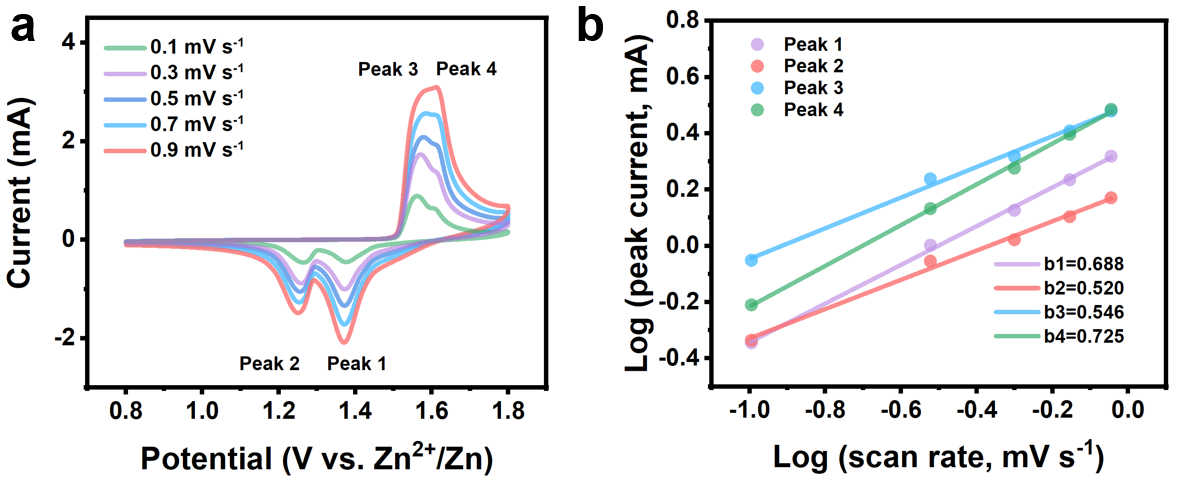


**Figure S26**. a) CV curves of pristine MnO_2_ at different scan rates. b) Log *i* *vs* log *v* plots of pristine MnO_2_.


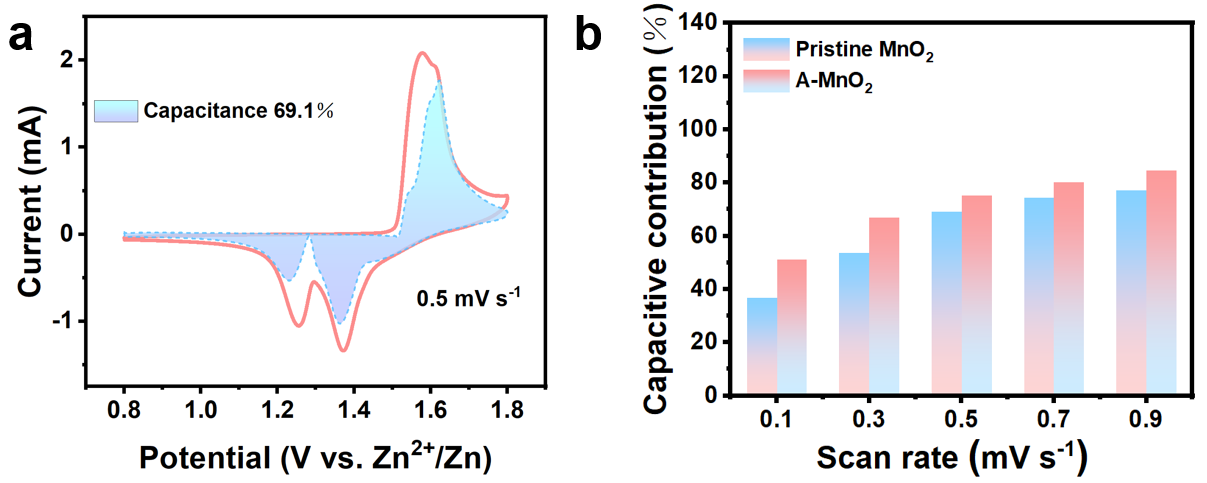


**Figure S27**. a) The capacitive contribution of pristine MnO_2_ at a scan rate of 0.5 mV s^−1^. b) Capacitive contributions of pristine MnO_2_ and A-MnO_2_ cathodes.


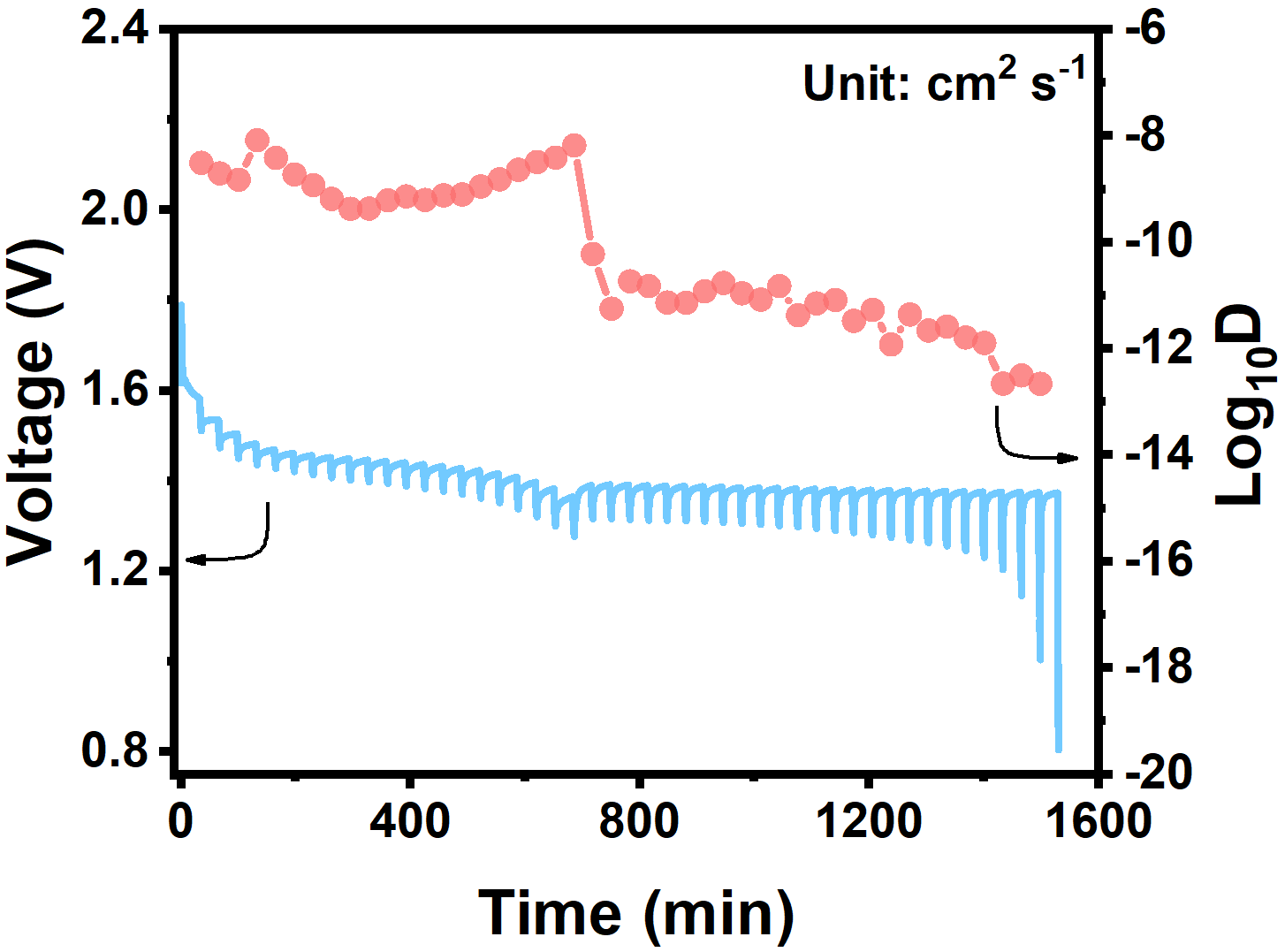


**Figure S28**. GITT profiles and the corresponding ion diffusion coefficient of pristine MnO_2_

**References**

[1] G. Kresse, J. Furthmüller, *Comput. Mater. Sci.* **1996**, 6, 15.

[2] J. P. Perdew, K. Burke, M. Ernzerhof, *Phys. Rev. Lett.* **1996**, 77, 3865.

[3] S. Grimme, *J. Comput. Chem.* **2006**, 27, 1787.

[4] G. Henkelman, B. P. Uberuaga, H. Jónsson, *J. Chem. Phys.* **2000**, 113, 9901.
